# WHSC1L1-mediated EGFR mono-methylation enhances the cytoplasmic and nuclear oncogenic activity of EGFR in head and neck cancer

**DOI:** 10.1038/srep40664

**Published:** 2017-01-19

**Authors:** Vassiliki Saloura, Theodore Vougiouklakis, Makda Zewde, Xiaolan Deng, Kazuma Kiyotani, Jae-Hyun Park, Yo Matsuo, Mark Lingen, Takehiro Suzuki, Naoshi Dohmae, Ryuji Hamamoto, Yusuke Nakamura

**Affiliations:** 1 Department of Medicine, Section of Hematology/Oncology, University of Chicago, Chicago, USA; 2 Department of Medicine, University of Chicago, Chicago, USA; 3 OncoTherapy Science Inc., Kawasaki, Japan; 4 Department of Pathology, University of Chicago, Chicago, USA; 5 Biomolecular Characterization Unit, RIKEN Center for Sustainable Resource Science, Saitama, Japan.; 6 Department of Surgery, University of Chicago, Chicago, USA.

## Abstract

While multiple post-translational modifications have been reported to regulate the function of epidermal growth factor receptor (EGFR), the effect of protein methylation on its function has not been well characterized. In this study, we show that WHSC1L1 mono-methylates lysine 721 in the tyrosine kinase domain of EGFR, and that this methylation leads to enhanced activation of its downstream ERK cascade without EGF stimulation. We also show that EGFR K721 mono-methylation not only affects the function of cytoplasmic EGFR, but also that of nuclear EGFR. WHSC1L1-mediated methylation of EGFR in the nucleus enhanced its interaction with PCNA in squamous cell carcinoma of the head and neck (SCCHN) cells and resulted in enhanced DNA synthesis and cell cycle progression. Overall, our study demonstrates the multifaceted oncogenic function of the protein lysine methyltransferase WHSC1L1 in SCCHN, which is mediated through direct non-histone methylation of the EGFR protein with effects both in its cytoplasmic and nuclear functions.

The epidermal growth factor receptor (EGFR) belongs to the well characterized family of receptor tyrosine kinases (RTK) and is known to play important roles in normal development as well as cancer pathogenesis through promotion of cell proliferation, invasion, migration, metastasis and angiogenesis^1^,^2^,^3^,^4^. It is primarily a cytoplasmic-membrane RTK activated by growth factor ligands, such as epidermal growth factor and amphiregulin, which induce homodimerization and/or heterodimerization of EGFR with other members of the same RTK family (HER2, HER3, HER4). This dimerization increases the intrinsic tyrosine kinase activity and subsequent autophosphorylation of C-terminal tyrosine (Y) residues, such as Y992, Y1068, Y1148, and Y1173 of EGFR. These phosphotyrosines function as docking sites for SH2-containing messenger molecules, which in turn activate the downstream pathways of RAS-RAF-MEK-ERK, PI3K-AKT-mTOR and STAT3, leading to DNA synthesis and cell proliferation, invasion and migration^5^,^6^,^7^,^8^.

Post-translational modifications of EGFR, such as phosphorylation, glycosylation and ubiquitination, are known to modulate the function of EGFR^6^,^7^,^9^,^10^,^11^,^12^. No reports though have described the functions of EGFR lysine methylation. More recently, EGFR arginine (R) methylation was reported to play significant roles in the regulation of EGFR function. Hsu *et al*.^13^ reported that PRMT5-mediated EGFR methylation at R1175 functions in a tumor suppressive manner; this methylation enhances Y1173 phosphorylation and augments its interaction with the SH2-domain-containing protein tyrosine phosphatase 1 (SHP1) which results in attenuation of the EGFR-ERK-dependent pathway. In this study, the authors have proposed the hypothesis that PRMT5-mediated methylation of R1175 enhances Y1173 EGFR phosphorylation and its interaction with SHP1, rather than activates ERK-signaling proteins SH2-domain-containing transforming protein (SHC) and growth factor receptor-bound protein 2 (GRB2). More recently, Liao *et al*.^14^ reported that PRMT1-mediated methylation of R198/200 in the extracellular domain of EGFR enhances its binding to EGF and its homodimerization, and activates both the RAS-RAF-MEK-ERK and the PI3K-AKT growth-signaling pathways in colon cancer cells.

EGFR is shuttled from the cell membrane to the nucleus after endocytosis as well as retrograde transport from the Golgi to the endoplasmic reticulum (ER)^15^. In addition, a putative nuclear localization signal (NLS) has been described within the juxtamembrane regions among the EGFR family^16^ which enables nuclear translocation of EGFR. Nuclear EGFR can also exit the nucleus through the exportin CRM1 (chromatin region maintenance 1), although specific nuclear export signals have not been identified yet^17^. Nuclear EGFR exists in a full-length EGFR form and maintains its tyrosine kinase activity. In the nucleus, it interacts with E2F1, RNA helicase A, DNA-dependent protein kinase (DNA-PK), signal transducer and activator of transcription 3 (STAT3), STAT5 and proliferating cell nuclear antigen (PCNA)^18^,^19^. Through such protein interactions, nuclear EGFR functions as a transcription co-factor to activate gene promoters and regulate multiple biological processes, such as cell proliferation, DNA repair and replication, chemo- and radioresistance^20^,^21^. Nuclear EGFR also associates and phosphorylates the chromatin-bound form of PCNA, it stabilizes it, and leads to DNA replication and DNA repair^22^. In addition, high expression of nuclear EGFR is correlated with poor survival and chemoradiation resistance in head and neck, and esophageal cancer patients^23^,^24^. No studies have reported specific effects of post-translational modifications, and particularly methylation, on nuclear EGFR.

Wolf-Hirschhorn Syndrome Candidate 1-Like 1 (WHSC1L1) is a protein lysine methyltransferase which di-methylates lysine 36 on histone 3 (H3K36). It has two main isoforms, the long WHSC1L1 (1437aa) and the short WHSC1L1 (645aa) which lacks the enzymatic methyltransferase domain (SET, Suppressor of variegation 3–9, Enhancer of zeste and Trithorax domain). WHSC1L1 is amplified in multiple cancer types, including squamous cell lung cancer, breast cancer, bladder cancer, and squamous cell carcinoma of the head and neck (SCCHN)^25^,^26^,^27^. We previously reported that WHSC1L1 knockdown led to cell cycle arrest at the G2/M phase of bladder and lung adenocarcinoma cancer cells and found CCNG1 (cyclin G1) and NEK7 to be potential downstream genes of WHSC1L1^28^. Furthermore, we recently showed that WHSC1L1 was significantly overexpressed in SCCHN tissues compared to normal epithelium, and its siRNA-mediated knockdown significantly decreased cell viability in SCCHN cells^29^.

Mounting evidence over the past decade supports that protein methylation regulates oncogenesis not only through histone modification, but also through methylation of non-histone substrates^30^,^31^. While we found that WHSC1L1 functions as an oncogene by transcriptionally upregulating *CDC6* and *CDK2* through H3K36 di-methylation in SCCHN cells^29^, we further attempted to assess whether the oncogenic activity of WHSC1L1 is also mediated through a non-histone protein substrate(s). In this study, we show that WHSC1L1 mono-methylates lysine 721 in the tyrosine kinase domain of EGFR, and that this methylation enhances activation of its downstream RAS-RAF-MEK-ERK cascade, even without epidermal growth factor stimulation. We also show that WHSC1L1-mediated EGFR mono-methylation affects the function of nuclear EGFR by enhancing its interaction with PCNA, and that this may be a novel mechanism to enhance DNA synthesis and S phase progression. These findings may have significant clinical implications and provide the scientific rationale for further investigation of WHSC1L1 inhibition as a novel treatment approach in patients with SCCHN.

## Results

### WHSC1L1 mono-methylates EGFR at lysine K721 in its tyrosine kinase domain both *in vitro* and *in vivo*

**Figure 1:**
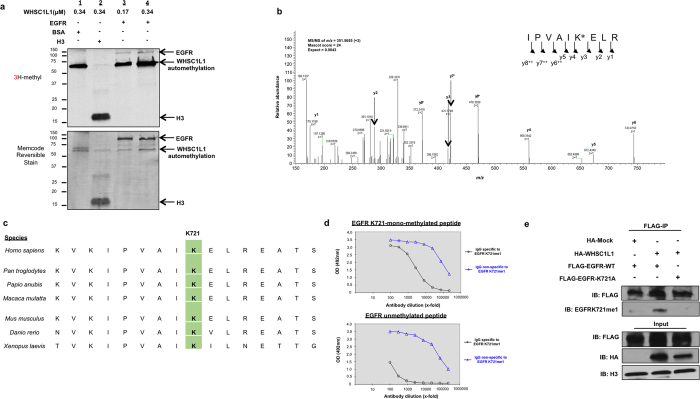
WHSC1L1 methylates EGFR at lysine K721 *in vitro* and *in vivo*. (**a**) WHSC1L1 methylates EGFR in a dose-dependent manner. *In vitro* methyltransferase assay of WHSC1L1 with recombinant EGFR. Recombinant EGFR was incubated with increasing amounts of WHSC1L1 in the presence of ^3^H-SAM. Bovine serum albumin was used as a negative control and recombinant histone H3 as a positive control. The reactants were analyzed by SDS-PAGE followed by fluorography for 3 days (upper panel). The PVDF transfer membrane was then stained with MemCode reversible protein stain to visualize the total protein (lower panel). (**b**) The MS/MS spectrum corresponding to the mono-methylated EGFR fragment IPVAIKEL (716-723). The mono-methylation corresponds to lysine (K) at position 721 within the tyrosine kinase domain of EGFR. MS/MS score, Mascot ion score and Expectation value in Mascot Database search results are shown. (**c**) Aminoacid sequence alignment of human EGFR. The IPVAIKEL sequence which includes lysine K721 is located within the tyrosine kinase domain of EGFR and is preserved from *Homo sapiens* to *Xenopus laevis*. (**d**) Evaluation of the specificity of anti-mono-methylated K721 EGFR antibody using enzyme-linked immunosorbent assay (ELISA). Y-axis represents ELISA optical density (OD) units read at 492 nm. ELISA plates coated with the mono-methylated K721 EGFR peptide versus the unmodified EGFR peptide were incubated with the primary rabbit antisera for 16 h at 4oC. Detection was performed using a secondary rabbit antibody conjugated with horseradish peroxidase. Primary rabbit antisera were examined after dual selection against the modified versus the unmodified peptides. (**e**) 293T cells were cotransfected with FLAG-EGFR-WT or FLAG-EGFR-K721A and HA-Mock or HA-WHSC1L1. Immunoprecipitation with an anti-FLAG antibody was performed and immunoprecipitates were blotted with anti-mono-methylated K721 EGFR, anti-FLAG and anti-HA antibodies.

To investigate whether WHSC1L1 methylates any substrates other than histone H3, we performed *in vitro* methyltransferase assays using a proprietary library of recombinant oncogenic or tumor-suppressor proteins that are known to be important in SCCHN oncogenesis. This initial screening revealed EGFR as a potential substrate of WHSC1L1. *In vitro* methyltransferase assay with increasing amounts of WHSC1L1 revealed dose-dependent EGFR methylation (Fig. 1a), further supporting WHSC1L1-mediated EGFR methylation. In order to confirm the EGFR methylation by WHSC1L1 and to identify a methylated amino-acid residue(s), we performed mass spectrometry analysis of the *in vitro* methylated EGFR and found that WHSC1L1 mono-methylated lysine K721 in the tyrosine kinase domain of EGFR (Fig. 1b). Given the high conservation of lysine K721 among various species from *Xenopus laevis* to *Homo sapiens* (Fig. 1c), we presumed that mono-methylation of K721 may play a significant role in the functional regulation of EGFR.

We subsequently generated an antibody specific to K721 mono-methylated EGFR using a synthetic 13 amino-acid peptide including the mono-methylated K721 (CVAIK(Me)ELREATSP). We first examined its affinity and specificity using enzyme-linked immunosorbent assays (Fig. 1d). To assess whether this methylation occurs in living cells and to further confirm the specificity of this antibody, we constructed FLAG-tagged EGFR vectors with and without substitution of lysine K721 for alanine (FLAG-EGFR-WT and FLAG-EGFR-K721A, respectively). We then cotransfected 293T cells with HA-Mock and FLAG-EGFR-WT, HA-WHSC1L1 and FLAG-EGFR-WT or HA-WHSC1L1 and FLAG-EGFR-K721A vectors. Western blot analysis of FLAG-immunoprecipitates using the anti-K721-mono-methylated EGFR antibody revealed a positive signal specifically in 293T cells cotransfected with HA-WHSC1L1 and FLAG-EGFR WT, but this signal was nearly completely abolished in cells cotransfected with HA-WHSC1L1 and FLAG-EGFR-K721A, or HA-Mock and FLAG-EGFR-WT (Fig. 1e). EGFR K721 mono-methylation was also confirmed with mass spectrometry analysis of FLAG-immunoprecipitates of 293T cells cotransfected with FLAG-EGFR-WT and HA-WHSC1L1 (Supplementary Figure 1). These results support that WHSC1L1 mono-methylates EGFR at lysine K721 both *in vitro* and *in vivo*.

### Correlation of K721 mono-methylated EGFR with WHSC1L1 and clinical parameters in patients with SCCHN

**Figure 2:**
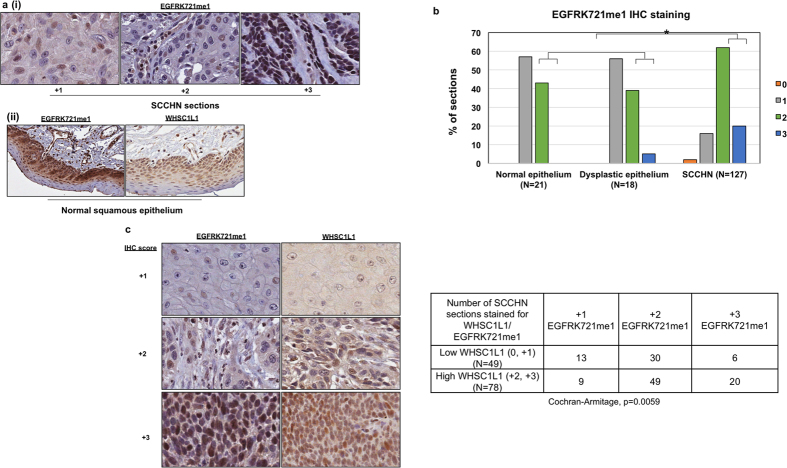
Immunohistochemical (IHC) staining of EGFRK721me1 in SCCHN tissues and correlation with WHSC1L1 staining. (**a**) (**i**) Representative examples of EGFRK721me1 IHC staining (+1, +2, +3 scores) in SCCHN tissue sections. Staining of EGFRK721me1 is mostly observed in the nucleus, with mild staining in the cytoplasm (40x). (**ii**) Representative example of EGFRK721me1 staining in normal squamous epithelium. Staining was mostly observed in the cells of the basal layer of the epithelium, corresponding to the staining of WHSC1L1 (20x). (**b**) Histogram of immunohistochemistry scores of EGFRK721me1 in normal, dysplastic epithelium and SCCHN tissue sections. The ratio of +2/+3 EGFRK721me1-positive staining samples was statistically significantly higher in SCCHN tissues compared to normal and dysplastic squamous epithelium (Cochran-Armitage test, *p < 0.001). (**c**) Representative examples of EGFRK721me1 and concordant WHSC1L1 staining in SCCHN sections from 3 different patients (40x). A table with the cumulative IHC score results of SCCHN sections (N = 127) staining for WHSC1L1 and EGFRK721me1 is also shown (Cochran-Armitage test, p = 0.0059).

We previously reported that WHSC1L1 is significantly overexpressed in SCCHN samples compared to normal epithelium^29^. To assess the pattern and levels of EGFRK721me1 in SCCHN, we performed immunohistochemical staining of 127 SCCHN samples, 21 normal squamous and 18 dysplastic epithelial samples. EGFRK721me1 was mostly localized in the nucleus, though weak staining was also observed in the cytoplasm of SCCHN cells (Fig. 2(a) (i)). 62% of SCCHN samples had moderate staining for EGFRK721me1 (+2) and 20% had strong staining (+3), while 16% stained weakly (+1). 38% of dysplastic samples had moderate staining and 5% of them stained strongly, while 55% of samples stained weakly. The majority of normal squamous epithelial samples (57%) stained weekly, while 43% of them showed moderate staining for EGFRK721me1. In the normal and dysplastic squamous epithelium, staining was observed mostly in the basal layer of cells. Interestingly, in normal squamous epithelium, both EGFRK721me1 and WHSC1L1 staining were observed only in the basal layer (Fig. 2(a)(**ii**)). The ratio of +2/+3 EGFRK721me1-positive staining samples was statistically significantly higher in SCCHN tissues compared to normal and dysplastic squamous epithelium (Cochran-Armitage, p < 0.001) (Fig. 2b). The correlation between WHSC1L1 and EGFRK721me1 protein levels was also examined and was found to be significant (representative examples and table in Fig. 2c) (Cochran-Armitage, p = 0.0059), further supporting the *in vivo* methylation of EGFR by WHSC1L1. No associations between EGFRK721me1 levels and various clinical parameters, including overall and progression-free survival, were found to be statistically significant (Supplementary Table 1, Supplementary Figure 2). Given the absence of correlations with survival and the fact that EGFRK721me1 levels increased significantly in the transition from normal squamous epithelium to dysplasia and SCCHN, EGFRK721me1 may be important in the initial stage of head and neck oncogenesis. This finding is in accordance with our previous report for WHSC1L1^29^.

### WHSC1L1-mediated mono-methylation of EGFR at lysine K721 enhances activating phosphorylation marks of EGFR

**Figure 3:**
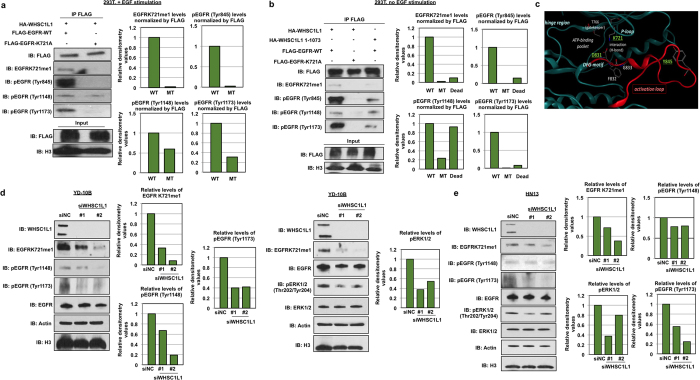
WHSC1L1-mediated mono-methylation of EGFR at lysine K721 enhances activating phosphorylation marks of EGFR independently of EGF stimulation and WHSC1L1 knockdown decreases phospho-EGFR and phospho-ERK in SCCHN cells. (**a**) 293T cells were cotransfected with HA-WHSC1L1 and FLAG-EGFR-WT versus FLAG-EGFR-K721A. After 48 h of serum starvation and EGF stimulation, FLAG-immunoprecipitates were blotted for phospho-EGFR marks. Densitometry of immunoblots from this experiment is also shown. (**b**) 293T cells were cotransfected with HA-WHSC1L1 and FLAG-EGFR-WT, HA-WHSC1L1 and FLAG-EGFR-K721A, or an enzyme dead variant of WHSC1L1 (HA-WHSC1L1 1-1073) and FLAG-EGFR-WT. Cells were serum starved for 48 h and FLAG-immunoprecipitates without EGF stimulation were blotted for phospho-EGFR marks. Densitometry of immunoblots from this experiment is also shown. (**c**) 3D structural representation of the tyrosine kinase domain of EGFR in its “DFG-in” form (Protein Data Bank, entry 2GS7). (**d**) Effect of WHSC1L1 (nuclear extract) knockdown on phospho-EGFR marks, EGFR K721 mono-methylation and phospho-ERK1/2 levels (cytoplasmic extract) in YD-10B cells assessed by Western blotting. Actin and H3 were blotted as loading controls. Densitometry of immunoblots from each experiment is also shown. Data were obtained from two separate experiments. (**e**) Effect of WHSC1L1 (nuclear extract) knockdown on phospho-EGFR marks, EGFRK721 mono-methylation and phospho-ERK1/2 levels (cytoplasmic extract) in HN13 cells assessed by Western blotting. Actin and H3 were blotted as loading controls. Densitometry of immunoblots from this experiment is also shown. Data were obtained from the same experiment.

Several lines of evidence support that methylation of a lysine residue in a protein may affect other post-translational modifications at neighboring or distant amino-acid residues^30^. To examine the effect of K721 methylation on the phosphorylation of EGFR protein, we cotransfected 293T cells, which have low endogenous expression of WHSC1L1 and EGFR, with HA-WHSC1L1 and FLAG-EGFR-WT or with HA-WHSC1L1 and FLAG-EGFR-K721A, and kept the cells in a serum starved condition for 48 h. Then we stimulated the cells with EGF for 10 min and performed immunoprecipitation using an anti-FLAG antibody. The FLAG-immunoprecipitates were blotted for the analysis of phospho-Y845, phospho-Y1068, phospho-Y1148 and phospho-Y1173 EGFR, which represent the activated form of EGFR. Although we observed strong signals for phospho-Y845, phospho-Y1148 and phospho-Y1173 EGFR in 293T cells transfected with FLAG-EGFR-WT, these phosphorylation signals were decreased in 293T cells transfected with FLAG-EGFR-K721A (Fig. 3a). No difference in phospho-Y1068 EGFR was observed between the two conditions. These results indicate that K721 EGFR mono-methylation seems to be critically important for Y845, Y1148, and Y1173 phosphorylation of EGFR.

To assess whether EGFR K721 mono-methylation enhances the activating phosphorylation marks of EGFR independently of EGF stimulation, we cotransfected 293T cells with HA-WHSC1L1 and FLAG-EGFR-WT, HA-WHSC1L1 and FLAG-EGFR-K721A, or an enzyme dead variant of WHSC1L1 (HA-WHSC1L1 1-1073) and FLAG-EGFR-WT. Cells were serum starved as above for 48 h, but not stimulated by EGF before FLAG-immunoprecipitation. Results showed that, even in the absence of EGF stimulation, the cells transfected with HA-WHSC1L1 and FLAG-EGFR-WT revealed higher levels of phospho-Y845, phospho-Y1148 and phospho-Y1173 EGFR, while these phosphorylation marks were absent or diminished in the cells transfected with a combination of HA-WHSC1L1 and FLAG-EGFR-K721A, or with HA-WHSC1L1 1-1073 and FLAG-EGFR-WT (Fig. 3b). These results indicate that WHSC1L1-mediated EGFR K721 mono-methylation may play a critical role in EGFR activation even without EGF stimulation.

The above results were also supported by three-dimensional structural prediction analysis of the activation loop of EGFR (Fig. 3c)^32^. Lysine K721 is located in the tyrosine kinase domain of EGFR and interacts with aspartic acid at position 831 (D831) either directly with a polar hydrogen bond or salt bridge, or indirectly by binding to the phosphate group of the ATP molecule. Based on this prediction model, methylation of K721 could cause disruption of its polar interaction with the aspartic acid at position 831. This could displace the activation loop where the tyrosine at position 845 (Y845) is located which is critical for the enzymatic activity of EGFR (Fig. 3c). Based on the above, and given that knockdown of WHSC1L1 has been previously shown to cause growth suppression of SCCHN cells^29^, we postulated that mono-methylation of K721 may lead to activation of the enzymatic activity of EGFR through augmentation of EGFR tyrosine phosphorylation and this hypothesis was indeed supported by the aforementioned experiments.

To further examine the effect of WHSC1L1-mediated EGFR K721 mono-methylation on endogenous phospho-Y1148 and phospho-Y1173 EGFR marks in SCCHN cell lines, we performed siRNA-mediated knockdown of WHSC1L1 with two different WHSC1L1-specific siRNAs in two SCCHN cell lines, YD-10B and HN13, which endogenously overexpress wild-type WHSC1L1 and EGFR. After 48 hours of serum starvation and subsequent EGF stimulation (10 min), cellular extracts were obtained and blotted for phospho-Y1148 and phospho-Y1173 EGFR, total EGFR and EGFR K721 mono-methylated levels. Knockdown of WHSC1L1 led to a decrease in phospho-Y1148, phospho-Y1173 EGFR, and EGFR K721 mono-methylated levels in both YD-10B and HN13 cells, while no change was observed in total EGFR protein levels (Fig. 3d). Concordantly, given that the RAS-RAF-MEK-ERK module is activated through phospho-Y1148 and phospho-Y1173 EGFR, pERK1/2 levels were evaluated and found to be decreased in the WHSC1L1-depleted samples in both YD-10B and HN13 cells, while total ERK protein levels were unchanged (Fig. 3e). These results support that suppression of WHSC1L1-mediated EGFR K721 mono-methylation leads to reduction of EGFR activity as well as suppression of EGFR’s downstream signaling pathway involving RAS, RAF, MEK and ERK.

### WHSC1L1 interacts with nuclear EGFR and potentiates its interaction with PCNA through EGFR K721 mono-methylation in the nucleus of SCCHN cells

**Figure 4:**
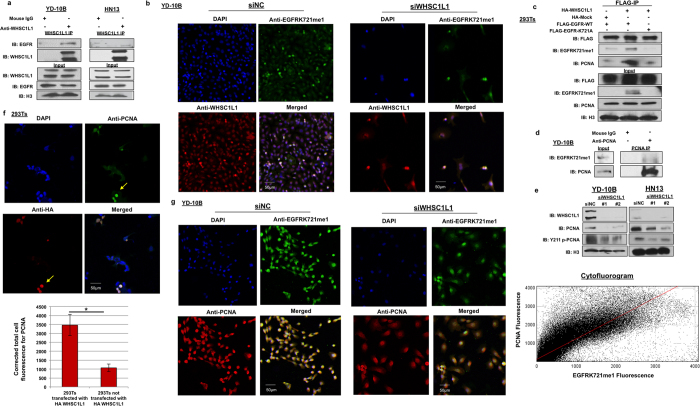
WHSC1L1 interacts with nuclear EGFR and potentiates its interaction with PCNA through EGFR K721 mono-methylation in the nucleus of SCCHN cells. (**a**) WHSC1L1 interacts with nuclear EGFR in SCCHN cells. Nuclear extracts from YD-10B and HN13 cells were obtained and immunoprecipitated using a WHSC1L1 specific antibody. Immunoprecipitates were blotted for EGFR. Expression of WHSC1L1 and EGFR was confirmed in YD-10B and HN13 nuclear extracts (input). H3 was used as a loading control. (**b**) WHSC1L1 expression correlates positively with EGFR K721 mono-methylation levels in the nucleus of YD-10B cells. Immunocytochemistry was performed in YD-10B cells treated with siNC or siWHSC1L1. Cells were stained with DAPI, anti-EGFRK721me1 and anti-WHSC1L1 antibodies. Results shown in 10x magnification. (**c**) WHSC1L1-mediated EGFR K721 mono-methylation enhances nuclear EGFR’s interaction with PCNA. 293T cells were cotransfected with FLAG-EGFR-WT and HA-Mock or HA-WHSC1L1, and FLAG-EGFR-K721A and HA-WHSC1L1 for 48 h. Protein extracts were sonicated and immunoprecipitated using a FLAG-antibody. FLAG-immunoprecipitates were immunoblotted for PCNA. (**d**) K721 mono-methylated EGFR interacts with PCNA in the nucleus of SCCHN cells. Nuclear extracts were obtained from YD-10B cells and PCNA immunoprecipitates were blotted for mono-methylated K721 EGFR. (**e**) siRNA-mediated WHSC1L1 knockdown, PCNA and Y211 PCNA phosphorylation levels in YD-10B and HN13 cells. Nuclear extracts were blotted for WHSC1L1, PCNA and Y211 PCNA. H3 was used as a loading control. (**f**) 293T cells were cotransfected with HA-WHSC1L1 and FLAG-EGFR-WT and immunocytochemistry was performed using anti-HA and anti-PCNA specific antibodies. HA-WHSC1L1 transfected (yellow arrows) and untransfected 293T cells are shown in correlation with PCNA levels. A representative histogram of corrected total cell fluorescence for PCNA in HA-WHSC1L1 transfected versus untransfected 293T cells is shown (Student’s t-test, p = 0.0019). Fluorescence intensity was analyzed using the Image J software and corrected total cell fluorescence was calculated as follows: Integrated density − (area of selected cell × mean fluorescence of background). (**g**) Correlation of PCNA and EGFRK721me1 levels. YD-10B cells were treated for 72 h with siNC versus siWHSC1L1 and plated in glass slide chambers. Immunocytochemistry was performed for PCNA and EGFR K721 mono-methylation. Results shown in 10x magnification. Cytofluorogram using the JacoP software is also presented (Pearson’s correlation co-efficient rho = 0.856, p < 0.0001).

Given that WHSC1L1 is mostly localized in the nucleus, we assessed whether WHSC1L1 interacts with EGFR in the nucleus of SCCHN cells. First, WHSC1L1 was immunoprecipitated from nuclear extracts of 2 SCCHN cell lines, YD-10B and HN13, using a WHSC1L1-specific antibody. Immunoblotting of WHSC1L1-immunoprecipitates with an anti-EGFR antibody indicated coimmunoprecipitation of WHSC1L1 and EGFR in the nucleus of SCCHN cells (Fig. 4a). This experiment indicates that WHSC1L1 interacts with EGFR in the nucleus of SCCHN cells.

To examine the correlation between WHSC1L1 levels and K721 mono-methylated EGFR levels in the nucleus of SCCHN cells, we performed immunocytochemistry for WHSC1L1 and K721 mono-methylated EGFR in YD-10B cells, and found a statistically significant correlation between WHSC1L1 and K721 mono-methylated EGFR (Pearson correlation co-efficient rho = 0.946, p < 0.0001) (Fig. 4b, Supplementary Figure 3).

Because the nuclear functions of EGFR are mostly known to be mediated through its interactions with nuclear proteins, and taking into consideration that protein methylation may affect the interaction between proteins^30^, we hypothesized that WHSC1L1-mediated K721 EGFR mono-methylation may affect the interaction of nuclear EGFR with its interacting proteins in the nucleus. Hence, 293T cells were cotransfected with FLAG-EGFR-WT and HA-Mock, FLAG-EGFR-WT and HA-WHSC1L1, or with FLAG-EGFR-K721A and HA-WHSC1L1. Cell lysates were immunoprecipitated using a FLAG antibody and the immunoprecipitates were blotted with STAT5, STAT3, E2F1, RNA helicase A, DNA-PK and PCNA. Among them, PCNA was found to interact more intensely with FLAG-EGFR-WT in 293T cells transfected with HA-WHSC1L1, compared to cells transfected with FLAG-EGFR-K721A and HA-WHSC1L1 or to those transfected with FLAG-EGFR-WT and HA-Mock (Fig. 4c, Supplementary Figure 4). We further performed immunoprecipitation of PCNA and subsequently immunoblotted for K721 mono-methylated EGFR using nuclear extracts from YD-10B cells, and confirmed the interaction of K721 mono-methylated EGFR and PCNA in the nucleus of SCCHN cells (Fig. 4d).

Nuclear EGFR has been shown to interact with PCNA and phosphorylate it at tyrosine 211 (Y211)^22^. This phosphorylation enhances the protein stability of PCNA, augmenting its function in DNA replication. Based on the finding that K721 mono-methylated EGFR interacts with PCNA, we hypothesized that this interaction may lead to increased Y211 phosphorylation and thus increased stability of PCNA. To evaluate the effect of WHSC1L1 on PCNA levels, siRNA-mediated WHSC1L1 knockdown was performed in YD-10B and HN13 cells and results showed a decrease in both the quantity of total PCNA protein and its Y211 phosphorylation levels (Fig. 4e). To further assess the effect of WHSC1L1 on PCNA levels, 293T cells were cotransfected with HA-WHSC1L1 and FLAG-EGFR-WT, and after 48 hours immunocytochemistry was performed using anti-HA and anti-PCNA antibodies to examine a potential correlation between the levels of WHSC1L1 and PCNA proteins. Results revealed that 293T cells transfected with HA-WHSC1L1 had significantly higher PCNA staining compared to 293T cells without HA-WHSC1L1 (Fig. 4f). To evaluate whether mono-methylated K721 EGFR levels correlate positively with PCNA levels, YD-10B cells were treated with control siRNA or a WHSC1L1-specific siRNA, and we performed immunocytochemical analysis for PCNA and K721 mono-methylated EGFR. Expectedly, we found a statistically significant positive association between the amounts of PCNA and K721 mono-methylated EGFR (Fig. 4g), implying that WHSC1L1-mediated K721 mono-methylation of EGFR is associated with increased amounts of PCNA protein in the nucleus of SCCHN cells (Pearson’s correlation co-efficient rho = 0.856, p < 0.0001).

### WHSC1L1-mediated K721 mono-methylation of nuclear EGFR enhances DNA replication

**Figure 5:**
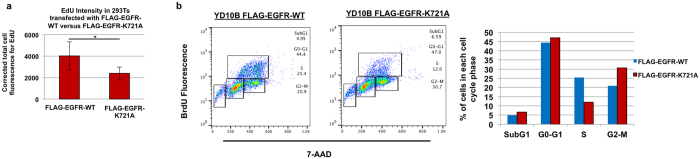
WHSC1L1-mediated K721 mono-methylation of nuclear EGFR enhances DNA replication. (**a**) 293T cells were transfected with HA-WHSC1L1 and FLAG-EGFR-WT versus HA-WHSC1L1 and FLAG-EGFR-K721A vectors. 24 h after transfection, cells were transferred to collagen-coated immunocytochemistry slides and were exposed to aphidicolin (5 μg/ml) for 24 h to synchronize the cell cycle. At 48 h from transfection, the cell cycle was released by removal of aphidicolin and 3 h post-release, when peak of the S phase is expected, the EdU imaging kit was initiated, while cells were also stained for FLAG expression. Fluorescence intensity was analyzed using the Image J software and corrected total cell fluorescence was calculated as follows: Integrated density – (area of selected cell x mean fluorescence of background). The average EdU fluorescence intensities were compared in 293T cells transfected with FLAG-EGFR-WT (average = 4022 ± 1310, standard error (SE)) versus FLAG-EGFR-K721A (average = 2395 ± 596 (SE)) and results are shown with a histogram (ANOVA test, *p = 0.0358). (**b**) YD-10B cells were transfected with FLAG-EGFR-WT versus FLAG-EGFR-K721A and at 24 h, they were exposed to aphidicolin (5 μg/ml) for cell cycle synchronization. After 24 h, aphidicolin was released and BrdU exposure was performed at 6 h post-aphidicolin release to capture the S-phase of YD-10B cells. Cells transfected with FLAG-EGFR-K721A showed a decrease in the S-phase percentage of cells compared to cells transfected with FLAG-EGFR-WT. The percentages of cells in each cell cycle phase are also shown in a histogram representative of this experiment. This experiment was duplicated.

Given that PCNA is necessary for DNA synthesis and entry of cells into the S phase and that WHSC1L1-mediated EGFR K721 mono-methylation is associated with increased PCNA levels, we sought to evaluate whether WHSC1L1-mediated K721 mono-methylation of EGFR affects entry of cells into the S phase. To this purpose, 293T cells were transfected with HA-WHSC1L1 and FLAG-EGFR-WT, or with HA-WHSC1L1 and FLAG-EGFR-K721A. 24 h after transfection, the cells were transferred to collagen-coated immunocytochemistry slides and exposed to aphidicholin (5ug/ml) for 24 h to arrest the cell cycle at the G1 phase. 3 hours later, the cells were released from the arrest by removing aphidicolin, incorporation of the modified thymidine analogue EdU was assessed by immunofluorescence, while cells were also stained for FLAG. The average EdU fluorescence intensities in 293T cells transfected with FLAG-EGFR-WT or those with FLAG-EGFR-K721A were compared (Fig. 5a). The average EdU fluorescence signals in FLAG-EGFR-WT-transfected cells were significantly higher than that of FLAG-EGFR-K721A-transfected cells (ANOVA test, p = 0.0358), indicating that EGFR K721 mono-methylation enhances DNA replication.

To validate this result, YD-10B cells which endogenously overexpress WHSC1L1 were transfected with FLAG-EGFR-WT or FLAG-EGFR-K721A and at 24 h they were exposed to aphidicolin for cell cycle synchronization and arrest at the G1 phase. After 24 h, the cells were released from the G1 cell cycle arrest by removing aphidicolin, and BrdU exposure was performed at 6 h post-aphidicolin release to capture the S-phase population of YD-10B cells. Cells transfected with FLAG-EGFR-K721A showed a decrease in the S-phase percentage of cells to 12%, compared to 25.4% of S-phase cells transfected with FLAG-EGFR-WT (Fig. 5b).

### WHSC1L1 knockdown sensitizes SCCHN cells to EGFR inhibition with erlotinib

**Figure 6:**
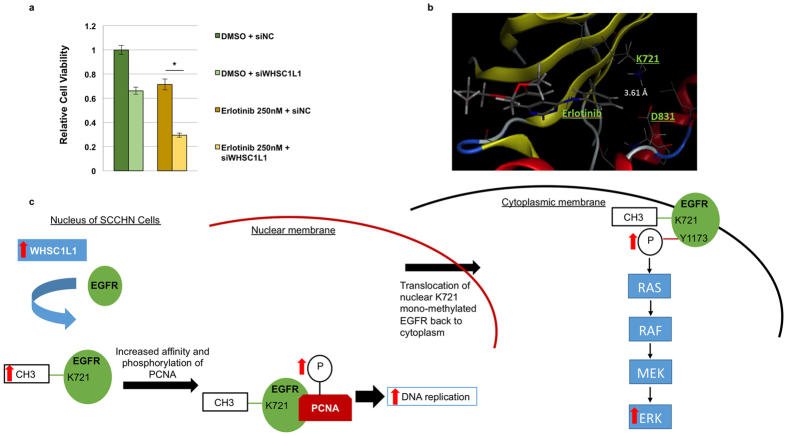
Effect of WHSC1L1 knockdown on sensitivity of SCCHN cells to erlotinib. (**a**) MTT assay of YD-10B cells treated with negative control siRNA versus WHSC1L1 specific siRNA for 48 h, and then exposed to erlotinib for 24 h (Student’s t-test, *p < 0.0001). All conditions are represented in quadruples. (**b**) 3D structural representation of the binding of erlotinib to the ATP-binding site of EGFR (modeled from Protein Data Bank, entry 4HJO). (**c**) Putative mechanism of action of WHSC1L1-mediated K721 mono-methylation of EGFR in the nucleus and cytoplasm of SCCHN cells.

To investigate whether WHSC1L1-mediated EGFR K721 mono-methylation could affect the sensitivity of SCCHN cells to EGFR inhibition, YD-10B cells with a high level of endogenous WHSC1L1 expression were treated with a WHSC1L1-specific siRNA or a control siRNA for 48 h, and then exposed to either DMSO or erlotinib for 24 h. MTT assay results showed a statistically significant decrease in cell viability from 71% in the cells pre-treated with control siRNA down to 29% in those pre-treated with WHSC1L1-specific siRNA (Fig. 6a). This result indicates that WHSC1L1-mediated K721 mono-methylation of EGFR induces resistance to erlotinib and that knockdown of WHSC1L1 is likely to sensitize SCCHN cells to EGFR inhibition.

To gain insight in the spatial relationship between the erlotinib binding site and the position of mono-methylated K721, a three-dimensional structural prediction analysis of the ATP-binding site was first pursued using the Molecular Operating Environment software (modeled from Protein Data Bank, entry 4HJO) (Fig. 6b). This analysis showed that, as erlotinib binds to the ATP-binding site of EGFR, the amino-group of K721 is neighboring to the phenyl-ring of erlotinib at a distance of 3.61 Angstrom. When K721 mono-methylation occurs, this distance is predicted to range from 2.19 to 4.79 Angstrom. In the context of this variability, K721 mono-methylation is expected to allow erlotinib to bind to the ATP-binding pocket without any van der Waals clash. This though presupposes that the K721 mono-methylation does not affect the position of other surrounding amino acids, such as D831, which is important for the binding of ATP. However, based on the result described above, it is possible that K721 mono-methylation may indeed influence the position of amino acids which are important for the binding of ATP, and may thus increase the binding affinity of ATP which competes with erlotinib for the ATP-binding pocket. This structural analysis may explain why WHSC1L1-mediated EGFR K721 mono-methylation may induce resistance to erlotinib.

## Discussion

In this study, we have showed that the protein lysine methyltransferase WHSC1L1 mono-methylates EGFR at lysine K721 in its tyrosine kinase domain, and leads to constitutive enhancement of the RAS-RAF-MEK-ERK pathway through augmentation of phospho-Y1148 and phospho-Y1173 in SCCHN cells even without EGF stimulation. We also showed that WHSC1L1-mediated K721 EGFR mono-methylation enhanced its interaction with PCNA. Given that EGFR maintains its tyrosine kinase activity in the nucleus, we showed that this interaction stabilized the PCNA protein and accelerated DNA replication in SCCHN cells. These findings are summarized in Fig. 5c.

An important point in the aforementioned findings is that we show that EGF stimulation is not required for the augmentation of the phosphorylation of Y1148 and Y1173. This may imply that, contrary to the known mechanisms of EGF-mediated EGFR homodimerization or the “classical” activating mutations of EGFR, such as in-frame exon 19 deletions and the L858R mutation^33^, which have been perceived as the main mechanisms of EGFR activation, WHSC1L1-mediated K721 EGFR mono-methylation may function as an activating mutation in the tyrosine kinase domain to elicit the constitutive activation of EGFR independent of EGF stimulation. An assessment of the mutation pattern of EGFR in the TCGA^25^,^26^ did not reveal any missense mutations at the K721 position of EGFR, supporting our finding that K721 mono-methylation may be critically important and may function as an activating event of the tyrosine kinase domain of EGFR. Furthermore, previous *in vitro* data^34^,^35^,^36^ have shown that inactivating mutations at K721 of EGFR reduce its tyrosine kinase activity, which further supports the importance of this residue in the tyrosine kinase activity of EGFR.

Additionally, to our knowledge this is the first report of the function of methylated nuclear EGFR. While Wang *et al*.^22^ previously published that nuclear, wild-type EGFR with intact tyrosine kinase activity phosphorylates PCNA at Y211 and stabilizes it, in this study we show that the methylation of nuclear EGFR at K721 affects its interaction with PCNA. More specifically, we show that WHSC1L1-mediated K721 mono-methylation of EGFR enhances its interaction with PCNA and increases its stability in the nucleus of SCCHN cells, leading to increased DNA replication. Taking the above into consideration, our findings support that WHSC1L1-mediated methylation of EGFR is not only necessary for its tyrosine kinase activity, but also for the protein interaction of nuclear EGFR with PCNA, illustrating the multifaceted, oncogenic functions of WHSC1L1.

Another important question that is raised in this study is the potential location where the K721 EGFR mono-methylation reaction takes place. Given that WHSC1L1 is a nuclear protein, we hypothesize that the K721 EGFR mono-methylation reaction takes place in the nucleus first. It is then possible that K721 mono-methylated EGFR is shuttled back from the nucleus to the cytoplasm. Reshuttling of nuclear EGFR back to the cytoplasm has previously been reported and considered possible through its interaction with CRM1^17^. Additionally, previous work by Huo *et al*.^37^ showed that treatment of epidermoid carcinoma A431 cells and breast cancer MDA-MB468 cells with a tyrosine kinase inhibitor did not inhibit the nuclear translocation of EGFR, indicating that unphosphorylated EGFR may also exist in the nucleus. It is thus possible that the unphosphorylated nuclear EGFR is a methylation substrate of WHSC1L1 in SCCHN cell lines that overexpress this enzyme. A fraction of K721 mono-methylated nuclear EGFR may shuttle back to the cytoplasmic membrane where it activates the EGFR downstream cascade in an EGF-independent manner, while another fraction remains nuclear and interacts with PCNA to enhance cell cycle progression and DNA replication. Although one of the functions of non-histone protein methylation is its effect on the subcellular localization of proteins^30^, our results did not support such an effect of WHSC1L1-mediated K721 EGFR mono-methylation (Supplementary Figure 5). Specifically, nuclear/cytoplasmic fractionation of protein extracts from 293T cells transfected with HA-WHSC1L1 and FLAG-EGFR-WT or HA-WHSC1L1 and FLAG-EGFR-K721A did not show a differential effect of the FLAG-EGFR-K721A vector on the levels of EGFR in the nuclear versus the cytoplasmic fractions. Similarly, knockdown of WHSC1L1 did not have an effect on the total cytoplasmic amounts of EGFR (Fig. 3d,e) in SCCHN cell lines, supporting no effect of WHSC1L1 on the subcellular localization of EGFR. On the other hand, K721 mono-methylated EGFR was detected in both the nuclear and the cytoplasmic fractions of 293T cells, indicating shuttling of K721 mono-methylated EGFR between the nucleus and the cytoplasm. Concordantly, our immunohistochemical results showed that K721 mono-methylated EGFR is localized both in the nucleus as well as the cytoplasm of SCCHN cells, particularly in tissues with strong EGFR K721 mono-methylation (Fig. 2a,c).

A direct therapeutic implication that could stem from this study is the potential role of WHSC1L1 in mediating resistance to EGFR inhibition. It is possible that mono-methylation of EGFR K721 by WHSC1L1 may allosterically affect the ATP binding site of EGFR (Fig. 6b), enhancing the binding of ATP and thus hindering the effective inhibition of EGFR by tyrosine kinase inhibitors, such as erlotinib. Another possibility is that WHSC1L1 may induce resistance to EGFR inhibition through potentiation of the functions of nuclear EGFR, particularly through increased PCNA levels (Fig. 6c). Nuclear EGFR has been described as a major mediator of resistance to therapy with EGFR tyrosine kinase inhibitors and EGFR antibodies such as cetuximab, but no effective strategies to overcome this resistance have been established in the clinic yet^20^. These mechanisms could potentially account for the low therapeutic efficacy of EGFR inhibition in SCCHN^38^ and could provide rationale for the combination of EGFR and WHSC1L1 inhibition in patients with SCCHN and WHSC1L1 overexpression. Future directions and ongoing work by our group to further prove this hypothesis include correlations of WHSC1L1 and EGFRK721me1 levels of tumor tissues with response and survival metrics after treatment with cetuximab-based chemotherapy in patients with recurrent/metastatic SCCHN.

We have previously shown that WHSC1L1 is significantly overexpressed in SCCHN and that its knockdown decreased the cell viability of SCCHN cells^29^. We attempted to explore various possible mechanisms for its oncogenic function and found that its effects are multifaceted, including the epigenetic regulation of downstream genes necessary for S-phase cell cycle progression through di-methylation of H3K36^29^, enhancement of the EGFR-ERK signaling cascade in an EGF-independent manner and augmentation of the association of nuclear EGFR with its interacting protein PCNA. Taking the above into consideration, and that WHSC1L1 has overall low expression levels in normal tissues^28^, this protein lysine methyltransferase may represent an important target for drug development in SCCHN patients with aberrant expression of this enzyme.

## Materials and Methods

### Immunohistochemistry in head and neck cancer tissue microarrays

The expression pattern of WHSC1L1 in 127 SCCHN, 18 dysplastic and 21 normal epithelial tissue sections were examined by immunohistochemistry. SCCHN sections were derived from biopsies of patients with local or locoregionally advanced disease previous to treatment with either surgery with or without adjuvant chemoradiation, or definitive chemoradiation. Slides of paraffin-embedded squamous cell carcinoma tumor specimens, dysplastic and normal epithelial tissues were deparaffinized, rehydrated and sections were treated with antigen retrieval buffer (pH 6, S2367, DAKO, Carpinteria, CA) in a steamer for 20 min at 96 °C. Anti-EGFRK721me1 antibody (customized, Anaspec, dilution 1:300) was applied on tissue sections for 1 h incubation at room temperature. Following TBS wash, the antigen-antibody binding was detected with the Bond Refine polymer detection system (DS9800, Leica Biosystems, Wetzlar, Germany) and DAB + chromogen (DAKO, K3468). Tissue sections were briefly immersed in hematoxylin for counterstaining of the nucleus and were covered with cover glasses. An expert head and neck cancer pathologist and an additional reviewer blinded to clinical outcomes performed semi-quantitative analysis of EGFRK721me1 staining using a four-grade scale defined as follows: negative, grade 0; mild, grade +1; moderate, grade +2; and strong staining intensity, grade +3. The tissue samples were acquired with written informed consent from all the participating patients following the relevant protocol approval by the University of Chicago Institutional Review Board (IRB 12-2125 and IRB 12-2117). Accordingly, the above experimental procedure involving patient samples was conducted in accordance with the approved guidelines by the University of Chicago Institutional Review Board (IRB 12-2125 and IRB 12-2117).

### Cell culture and plasmid transfections

Squamous cell carcinoma cell lines HN13 and YD-10B were derived from patients with locoregionally advanced SCCHN and were kindly provided by Dr. Tanguy Seiwert (University of Chicago). Detailed characteristics of each cell line are shown in Supplementary Table 2. HN13 cells were maintained in DMEM medium with 10% fetal bovine serum, 1% penicillin/streptomycin, and 2 nM L-glutamine. YD-10B cells were maintained in RPMI medium, 10% fetal bovine serum, 1% penicillin/streptomycin and 2 nM L-glutamine. Human embryonic kidney 293T cells were maintained in DMEM medium with 10% fetal bovine serum and 1% penicillin/streptomycin. All cells were maintained at 37 °C in humid air with 5% CO2 condition. Cells were transfected with FuGENE HD (Roche Applied Science, Madison, WI) according to manufacturer’s protocols.

### Expression vector construction

An entire coding sequence of WHSC1L1 (GenBank NCBI Reference Sequence: NM_023034.1) was amplified from human testis cDNAs using KOD-Plus-Neo (TOYOBO, Osaka, Japan) DNA polymerase and cloned into pCAGGSn3FC vector between *NotI* and *XhoI* restriction enzyme sites (pCAGGS-WT WHSC1L1-HA). To prepare an enzyme-inactive WHSC1L1, the coding sequence of the SET domain was deleted from the entire coding sequence of WHSC1L1 (pCAGGS-WHSC1L1-HA 1-1073). For FLAG-EGFR-WT, the entire coding sequence of EGFR (GenBank NCBI Reference Sequence: NM_005228.3) was similarly amplified from human testis cDNAs as described above, using KOD-Plus-Neo DNA polymerase and cloned into pCAGGSn3FC vector between *NotI* and *XhoI* restriction enzyme sites (pCAGGS-WT EGFR-FLAG). To generate the mutant FLAG-EGFR-K721A vector, the KOD Xtreme kit was used with the following primers: EGFR-K721A-f1 5′-TCG CTA TCG CGG AAT TAA GAG AAG-3′, and EGFR-K721A-r1 5′-CGG GAA TTT TAA CTT TCT CAC CTT C-3′.

### Western blotting

Nuclear extracts were prepared using the Nuclear Extraction kit (Active Motif) to examine protein levels of WHSC1L1, PCNA, phospho-Y211 PCNA, EGFRK721me1 and histone H3, and cytoplasmic extracts using the NE-PER nuclear and cytoplasmic extraction kit (78833, ThermoFisher Scientific) were obtained to examine protein levels of cytoplasmic EGFR, phospho-Y1148 EGFR, phospho-Y1173 EGFR, ERK1/2, phospho-ERK1/2, EGFRK721me1 and ACTB. Sonicated 293T cell extracts were used to assess levels of FLAG-EGFR-WT, FLAG-EGFR-K721A, HA-WHSC1L1, EGFRK721me1, phospho-Y845 EGFR, phospho-Y1148 EGFR and phospho-Y1173 EGFR. Samples were prepared from the cells lysed with CelLytic M cell lysis reagent (Sigma-Aldrich) containing a complete protease inhibitor cocktail (Roche Applied Science), and whole cell lysates or immunoprecipitation (IP) products were transferred to nitrocellulose membrane. Protein bands were detected by incubating with horseradish peroxidase (HRP)-conjugated antibodies (GE Healthcare) and visualized with enhanced chemiluminescence (GE Healthcare). We declare that our blots were evenly exposed in each membrane and that the blots were not cropped to the bands. Primary antibodies were used as described in the “Antibodies” section. Band levels were quantified by the GS-800 calibrated imaging densitometer (Bio-Rad), as indicated.

### Immunocytochemistry

Cultured cells were fixed in 4% paraformaldehyde in 0.1 M phosphate buffer (pH 7.4) at room temperature for 30 min, permeabilized in 0.1% Triton X-100 (Sigma-Aldrich) for 3 min and blocked with 3% BSA for 1 h at room temperature. Fixed cells were incubated with primary antibodies overnight at 4 °C, then with Alexa Fluor-conjugated secondary antibodies (Molecular Probes, Life Technologies) and observed using a Leica confocal microscope (SP5 Tandem Scanner Spectral 2-Photon Confocal). Fluorescence intensity was analyzed using the Image J software and corrected total cell fluorescence was calculated as follows: Integrated density – (area of selected cell x mean fluorescence of background).

### Immunoprecipitation

Transfected 293T cells were lysed with CelLytic M cell lysis reagent (Sigma Aldrich) containing a complete protease and phosphatase inhibitor cocktail (Roche Applied Science). In a typical IP reaction, 300–500 μg of whole-cell extract was incubated with an optimum concentration of primary antibody. After the protein G beads had been washed three times in 1 ml of TBS buffer (pH 7.6), proteins that bound to the beads were eluted by boiling in Lane Marker Reducing Sample Buffer (Thermo Scientific).

### Dominant-negative experiments

293T cells were cultured for 24 h and transfected with a FLAG-EGFR-WT and HA-Mock expression vector, a FLAG-EGFR-WT and HA-WHSC1L1 expression vector or a FLAG-EGFR-K721A and HA-WHSC1L1 vector using FuGENE HD transfection reagent (Roche Applied Science). Cells were harvested 48 h after transfection and lysed with CelLytic M cell lysis reagent (Sigma-Aldrich) containing a complete protease inhibitor cocktail (Roche Applied Science). Samples were separated by standard SDS-PAGE and subsequently immunoblotted with anti-EGFRK721me1, anti-EGFR antibody, anti-phospho-EGFR antibodies (Y845, Y1148, Y1173), anti-FLAG, anti-HA and anti-H3 antibodies.

### Antibodies

Primary antibodies used were anti-WHSC1L1 (rabbit, 11345-1-AP, Proteintech, dilution used in WB: 1:5000), anti-WHSC1L1 (mouse, H00054904-B02P, Novus Biologicals, dilution used in ICC: 1:500), anti-FLAG (rabbit, F7425; Sigma-Aldrich; dilution used in WB: 1:20,000), anti-FLAG (mouse, M2; Sigma-Aldrich; dilution used in ICC: 1:2000), anti-HA (rabbit, H6908, Sigma-Aldrich; dilution used in WB: 1:2000), anti-EGFRK721me1 (rabbit, customized antibody, Anaspec, dilution used in WB: 1:5000, ICC: 1:100), EGFR (D38B1, rabbit, Cell Signaling Technology, dilution used in WB: 1:5000), phospho-Y845 EGFR (#2231, rabbit, Cell Signaling Technology, dilution used in WB: 1:1000), phospho-Y1148 EGFR (#4404, rabbit, Cell Signaling Technology, dilution used in WB: 1:1000), phospho-Y1173 EGFR (#4407 53A5, rabbit, Cell Signaling Technology, dilution used in WB: 1:1000), ERK (#9102, rabbit, Cell Signaling Technology, dilution used in WB: 1:1000), phospho-ERK1/2 (Thr202/Tyr204) (#9101, rabbit, Cell Signaling Technology, dilution used in WB: 1:1000), anti-PCNA (mouse, PC10: sc-56, Santa-Cruz Biotechnology, dilution used in WB: 1:5000, ICC: 1:100), anti-PCNA (rabbit, Abcam #18197, dilution used in ICC: 1:100), anti-Y211 PCNA (rabbit, Benthyl, dilution used in WB: 1:2000), anti-ACTB (mouse, A5441, Sigma-Aldrich, dilution used in WB: 1:5000), anti-H3 (rabbit, ab1791, Abcam, dilution used in WB: 1:50000).

### siRNA transfection

MISSION_ siRNA oligonucleotide duplexes were purchased from Sigma–Aldrich for targeting the human WHSC1L1 transcripts (SASI_Hs01_00082044 and SASI_Hs01_00082046). siNegative control (siNC), which consists of three different oligonucleotide duplexes, were used as control siRNAs (Cosmo Bio, Tokyo, Japan). The siRNA sequences are described in Supplementary Table 3. SCCHN cells were plated overnight in 10 cm plates and were transfected with siRNA duplexes (50 nM final concentration) using Lipofectamine RNAimax (Thermo Fisher Scientific) for 3 days at a confluence of ~50%.

### *In vitro* methyltransferase assays

For the *in vitro* methyltransferase assay, recombinant EGFR (0.3 μg/μL, 3.6 μM, Life Technologies, 90.5 kDa, #PV3872) was incubated with recombinant WHSC1L1 enzyme (BPS Bioscience, 0.62 μg/μL, 10.2 μM, 65 kDa) using 1 mCi S-adenosyl-L-[methyl-3H]-methionine (SAM; PerkinElmer) as the methyl donor in a mixture of 30 μL of methylase activity buffer (50 mM Tris-HCl at pH8.8, 10 mM dithiothreitol and 10 mM MgCl_2_) overnight at 30 °C. Proteins were separated on a 5–20% SDS-PAGE gel (Ready Gel; Bio-Rad), then transferred on a PVDF membrane and visualized by MemCode Reversible Stain (Thermo Scientific) and fluorography.

### EdU Imaging Kit

The Click-iT EdU Alexa Fluor 488 imaging kit was used to assess incorporation of EdU in 293T cells transfected with FLAG-EGFR-WT versus FLAG-EGFR-K721A. More specifically, 293T cells were transfected with FLAG-EGFR-WT and HA-WHSC1L1 or FLAG-EGFR-K721A and HA-WHSC1L1. After 24 h, cells were plated in glass-slides and incubated overnight. At 48 h of transfection, cells were labeled with EdU with exposure to 20 μM EdU labeling solution for 2 h. Then, fixation with 3.7% formaldehyde and permealization with 0.5% Triton X-100 were performed. Subsequently, detection of EdU was conducted per protocol using the Click-iT reaction cocktail (1xClick-iT EdU reaction buffer, CuSO4, Alexa Fluor azide (488) and 1x Click-iT EdU additive buffer) for 30 min protected from light at room temperature. Cells were then stained with anti-FLAG antibody (mouse, M2; Sigma-Aldrich; dilution used in ICC: 1:2000) and incubated overnight at 4 °C. Next day, cells were exposed to secondary anti-mouse antibody (Alexa Fluor 594) and then washed with PBS for 6 times. DAPI was then applied and slides were covered with coverslips. Visualization of cells was performed using a Leica confocal microscope (SP5 Tandem Scanner Spectral 2-Photon Confocal) and fluorescence intensity of EdU was analyzed using the Image J software in cells transfected with FLAG-EGFR-WT versus FLAG-EGFR-K721A vectors. Corrected total cell fluorescence was calculated as follows: Integrated density – (area of selected cell x mean fluorescence of background).

### Cell cycle analysis

The 5-bromo-20-deoxyuridine (BrdU) flow kit (BD Biosciences, San Jose, CA) was used to determine cell cycle kinetics. The assay was performed according to the manufacturer’s instructions. Briefly, YD-10B cells were seeded overnight in 10 cm tissue culture dishes and transfected with FLAG-EGFR-WT versus FLAG-EGFR-K721A described as above using FuGENE HD and OPTIMEM. After 24 h of transfection, medium was suctioned and cells were exposed to antibiotic-containing RPMI medium with aphidicolin (5 μg/mL) for 48 h to achieve cell cycle synchronization at the G0/G1 phase. At 48 h, cells were released from the cell cycle arrest by removing aphidicolin and were exposed to BrdU at 6 h after release from the cell cycle arrest (expected S phase for YD-10B cells). After 1 h of BrdU exposure, cells were fixed in a solution containing paraformaldehyde and saponin. Then samples were incubated with DNAase for 1 h at 37 °C and FITC-conjugated anti-BrdU antibody (dilution: 1:50) was added for 20 min at room temperature. Total DNA was stained with 7-amino-actinomycin D (7-AAD), followed by flow cytometric analysis.

### Mass spectrometry

Recombinant EGFR (Millipore, #14-531, 696aa-end, 86 kDa) was incubated with recombinant WHSC1L1 (BPS Bioscience, 0.62 μg/μL, 10.2 μM, 65 kDa) and 1mCi of “cold” S-adenosyl-methionine was used as a methyl donor in a mixture of 30 μL of methylase activity buffer (50 mM Tris-HCl at pH8.8, 10 mM dithiothreitol and 10 mM MgCl_2_) overnight at 30 °C. Reactants were separated on SDS-PAGE and stained with Simply Blue Safe Stain (Life Technologies). The excised EGFR bands were reduced in 10 mM tris (2-carboxyethyl) phosphine (Sigma-Aldrich) with 50 mM ammonium bicarbonate (Sigma-Aldrich) for 30 min at 37 °C and alkylated in 50 mM iodoacetamide (Sigma-Aldrich) with 50 mM ammonium bicarbonate for 45 min in the dark at 25 °C. Trypsin GOLD (Promega) solution was added with the enzyme to protein ratio at 1/50 (w/w) and incubated at 37 °C for 16 h. The resulting bands were extracted from gel fragments and separated on a 0.1 × 200mm home-made C18 column using 45 min linear gradient from 2% to 35% acetonitrile in 0.1% formic acid, with flow rate at 200 nl/min. The eluting protein fragments were analyzed with HCTultra ETD II mass spectrometer (Bruker Daltonics). The acquired MS and collision-induced dissociation (CID) MS/MS spectra were processed with Compass DataAnalysis 4.0 (Bruker Daltonics) and BioTools 3.1 software (Bruker Daltonics), followed by the database search on in-house Mascot server ver.2.3.01 (Matrix Science). We accepted the peptide identifications satisfying the Expectation value of <0.05 in Mascot Database search.

### Statistical analysis

Spearman rank correlation coefficients were calculated to investigate associations between WHSC1L1 and EGFRK721me1 expression levels (using the 4-point IHC scale). EGFRK721me1 expression levels were compared among tumor, dysplastic, and normal squamous epithelial samples using the Wilcoxon rank sum test and the Cochran-Armitage trend test. Clinicopathological correlations were performed in a retrospective manner. Expression levels were dichotomized as 0–1 vs. 2–3 and the association between IHC score and gender, age, smoking history, stage, T-stage, N-stage, grade, and HPV status were evaluated using Cochran-Armitage trend test or Fisher’s exact test. Cox regression was performed to examine whether EGFRK721me1 was prognostic for overall or progression-free survival. Student’s t-test or ANOVA were performed to compare continuous outcome variables in different groups from the cell line experiments. Values were presented as the mean plus or minus standard deviation. Significant difference between groups was noted when p-value was < 0.05.

## Additional Information

**How to cite this article**: Saloura, V. *et al*. WHSC1L1-mediated EGFR mono-methylation enhances the cytoplasmic and nuclear oncogenic activity of EGFR in head and neck cancer. *Sci. Rep.* **7**, 40664; doi: 10.1038/srep40664 (2017).

**Publisher's note:** Springer Nature remains neutral with regard to jurisdictional claims in published maps and institutional affiliations.

## Supplementary Material

Supplementary Information
