# Supplementary material for: WHSC1L1-mediated EGFR mono-methylation enhances the cytoplasmic and nuclear oncogenic activity of EGFR in head and neck cancer
Source: Sci Rep. 2017 Jan 19;7:40664. doi: 10.1038/srep40664 (PMC5244396; doi:10.1038/srep40664)
Supplement: Supplementary Information [file srep40664-s1.pdf]

Supplementary Data

Title: WHSC1L1-mediated EGFR mono-methylation enhances the cytoplasmic and nuclear oncogenic activity of EGFR in head and neck cancer.

Authors: Vassiliki Saloura, Theodore Vougiouklakis, Makda Zewde, Xiaolan Deng, Kazuma Kiyotani, Jae-Hyun Park, Yo Matsuo, Mark Lingen, Takehiro Suzuki, Naoshi Dohmae, Ryuji Hamamoto, Yusuke Nakamura

Supplementary Figure 1.

MS/MS analysis of FLAG-EGFR-WT immunoprecipitates from 293T cells cotransfected with HA-WHSC1L1 and FLAG-EGFR-WT.

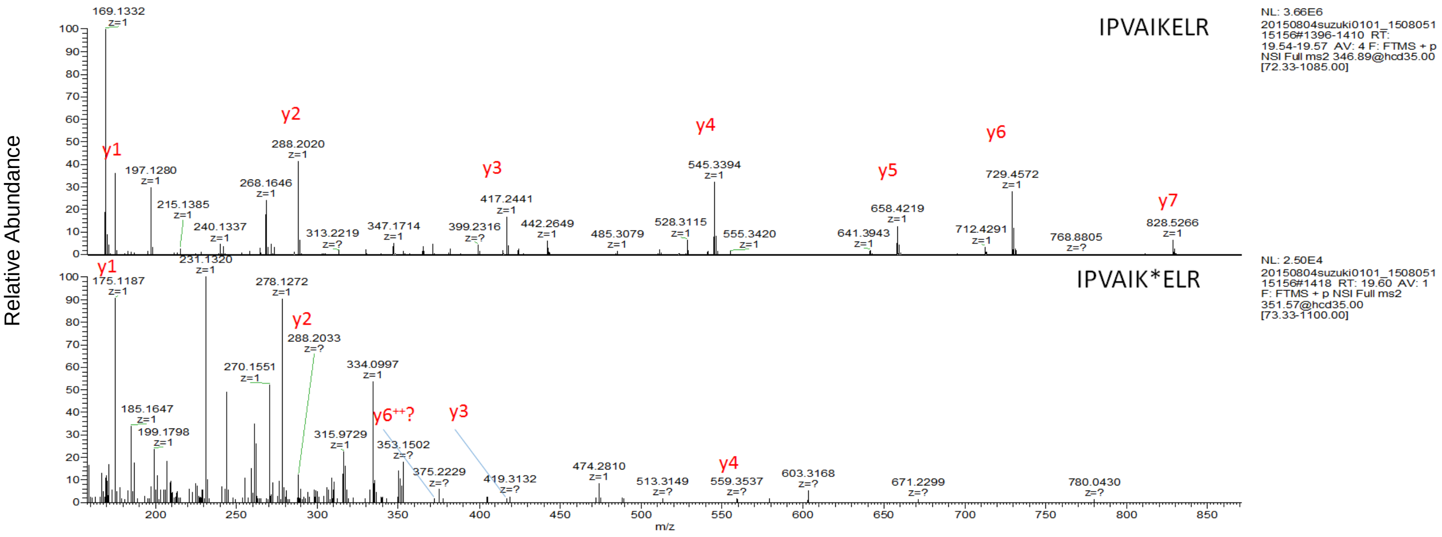

MSMS spectrum-full (in vivo: IPVAIKELR)

IPVAIKELR peptide MS/MS theoretical value

a	b	c"			x	y"	z
86.097	114.092	133.133	1 Ile	9	-	-	-
183.150	211.145	230.186	2 Pro	8	949.548	925.583	906.542
282.218	310.213	329.254	3 Val	7	852.495	828.531	809.490
353.255	381.250	400.291	4 Ala	6	753.427	729.462	710.421
466.339	494.334	513.375	5 Ile	5	682.390	658.425	639.384
594.434	622.429	641.470	6 Lys	4	569.306	545.341	526.300
723.477	751.472	770.513	7 Glu	3	441.211	417.246	398.205
836.561	864.556	883.597	8 Leu	2	312.168	288.204	269.162
-	-	-	9 Arg	1	199.084	175.120	156.078

IPVAIK*ELR+methyl peptide MS/MS theoretical value

a	b	c"			x	y"	z
86.097	114.092	133.133	1 Ile	9	-	-	-
183.150	211.145	230.186	2 Pro	8	963.564	939.599	920.558
282.218	310.213	329.254	3 Val	7	866.511	842.546	823.505
353.255	381.250	400.291	4 Ala	6	767.443	743.478	724.437
466.339	494.334	513.375	5 Ile	5	696.406	672.441	653.400
608.450	636.445	655.486	6 Lys	4	583.321	559.357	540.316
737.493	765.487	784.529	7 Glu	3	441.211	417.246	398.205
850.577	878.572	897.613	8 Leu	2	312.168	288.204	269.162
-	-	-	9 Arg	1	199.084	175.120	156.078

Supplementary Figure 2. Recurrence-free survival and overall survival of patients with locoregionally advanced SCCHN stratified by high (IHC score +2 and +3) or low (IHC score 0 and +1) levels of EGFRK721me1.

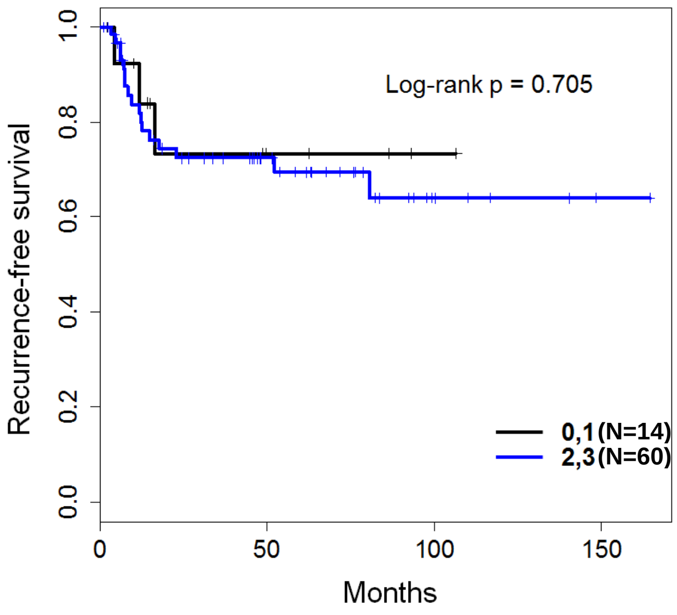

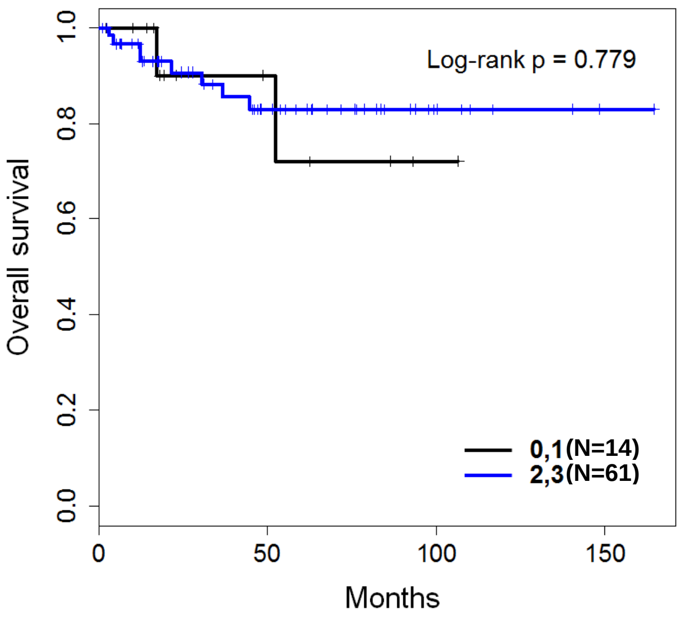

Supplementary Figure 3. Cytofluorogram showing the correlation between EGFRK721me1 and WHSC1L1 fluorescence in the nucleus of YD-10B cells treated with WHSC1L1 siRNA (Pearson's correlation co-efficient rho=0.946, p<0.0001).

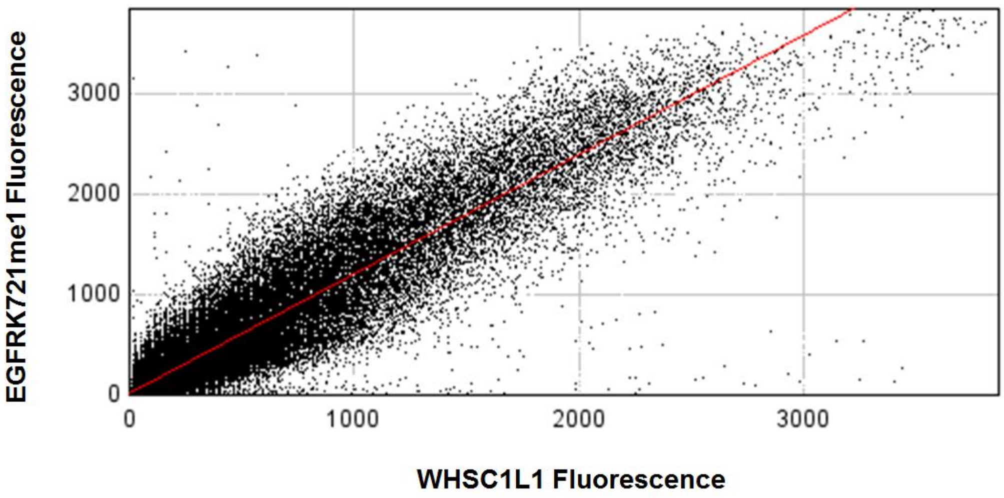

Supplementary Figure 4.

Densitometric analysis of Western blotting for exogenous FLAG-immunoprecipitation of FLAG-EGFR-WT versus FLAG-EGFR-K721A in 293T cells transfected with HA Mock or HA-WHSC1L1.

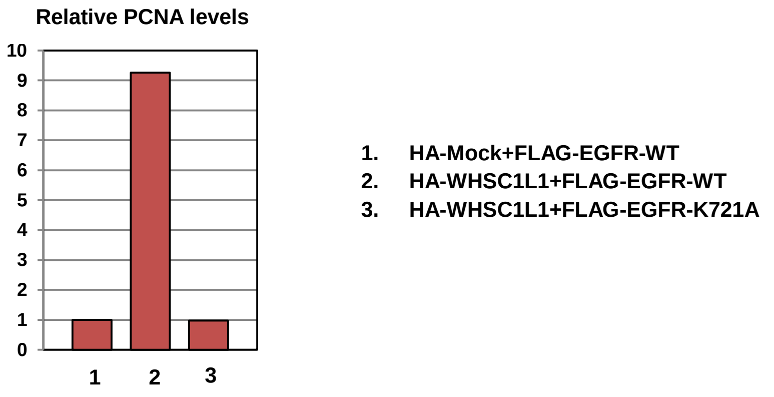

Supplementary Figure 5.

Effect of WHSC1L1-mediated K721 EGFR mono-methylation on the subcellular localization of EGFR. 293T cells were transfected for 48h with HA-WHSC1L1 and FLAG-EGFR-WT or HA-WHSC1L1 and FLAG-EGFR-K721A. Nuclear and cytoplasmic protein extracts were obtained and blotted for FLAG, EGFR and EGFRK721me1. H3 and actin were used as loading controls and to assess efficacy of nuclear/cytoplasmic separation.

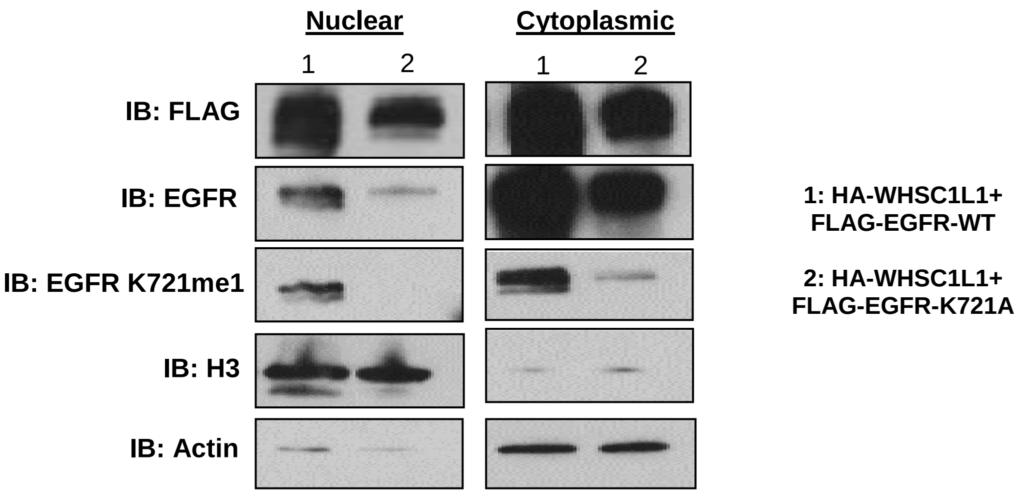

Supplementary Table 1.

Clinicopathological correlations of dichotomized EGFRK721me1 expression by IHC in locally and/or locoregionally advanced SCCHN.

**Clinicopathological Parameters**	**EGFRK721me1** **Low (0,+1) (n = 14)**	**High (+2,+3) (n = 68)**	***P* value**
Gender			0.77
Female	3	20	
Male	11	48	
Age (mean)	57	57.5	0.52
Primary site			0.64
Hypopharynx	6	11	
Larynx	9	17	
Tongue	10	9	
Tonsil	16	24	
Smoking status[1]			0.6
0py	4	12	
1-39py	5	28	
>=40py	4	19	
Grade[1]			0.064
Well differentiated	3	9	
Moderately differentiated	5	20	
Poorly differentiated	0	15	
Stage[1]			0.11
I	0	8	
II	0	1	
III	1	7	
IVA/B	13	49	
T[1]			0.47
T1	3	15	
T2	3	17	
T3	1	13	
T4	6	17	
N[1]			0.77
N0	4	17	
N1	0	9	
N2	9	37	
N3	1	4	
HPV status[1]			0.2
p16+	6	18	
p16-	6	42	

[1] Patients with missing data excluded

Supplementary Table 2.

Characteristics of SCCHN cell lines.

Cell name	TNM stage	Specimen site	Gender	HPV status
HN13	-	Oral tongue	-	-
YD-10B	-	Oral tongue	Male	HPV-negative

Supplementary Table 3.

SiRNA sequences for siNC, siWHSC1L1#1 and 2.

siRNA Name	Sequence
siNegative Control (siNC)	Target#1 Sense: 5’ AUCCGCGCGAUAGUACGUA 3’<br>Antisense: 5’ UACGUACUAUCGCGCGGAU 3’<br>Target#2 Sense: 5’ UUACGCGUAGCGUAAUACG 3’<br>Antisense: 5’ CGUAUUACGCUACGCGUAA 3’<br>Target#3 Sense: 5’ UAUUCGCGCGUAUAGCGGU 3’<br>Antisense: 5’ ACCGCUAUACGCGCGAAUA 3’
siWHSC1L1#1	Sense: 5’ CUCAUUGACUCCGCCAACA 3’<br>Antisense: 5’ UGUUGGCGGAGUCAAUGAG 3’
siWHSC1L1#2	Sense: 5’ CAGAAGAUCUUCCUCCACU 3’<br>Antisense: 5’ AGUGGAGGAAGAUCUUCUG 3’

Raw Data

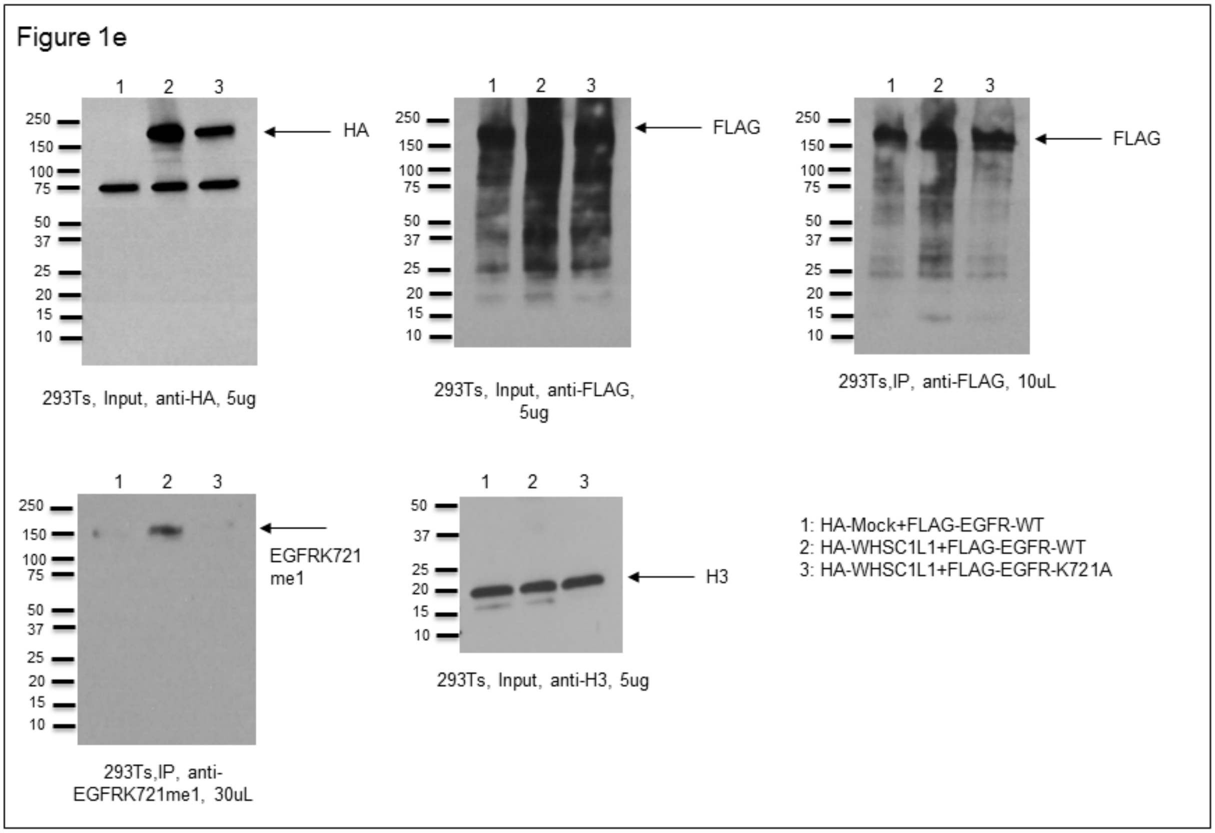
Figure 1e
1 2 3
250
150
100
75
50
37
25
20
15
10
HA
293Ts, Input, anti-HA, 5ug
FLAG
293Ts, Input, anti-FLAG, 5ug
FLAG
293Ts,IP, anti-FLAG, 10uL
EGFRK721 me1
293Ts,IP, anti-EGFRK721me1, 30uL
H3
293Ts, Input, anti-H3, 5ug
1: HA-Mock+FLAG-EGFR-WT
2: HA-WHSC1L1+FLAG-EGFR-WT
3: HA-WHSC1L1+FLAG-EGFR-K721A

Figure 3a

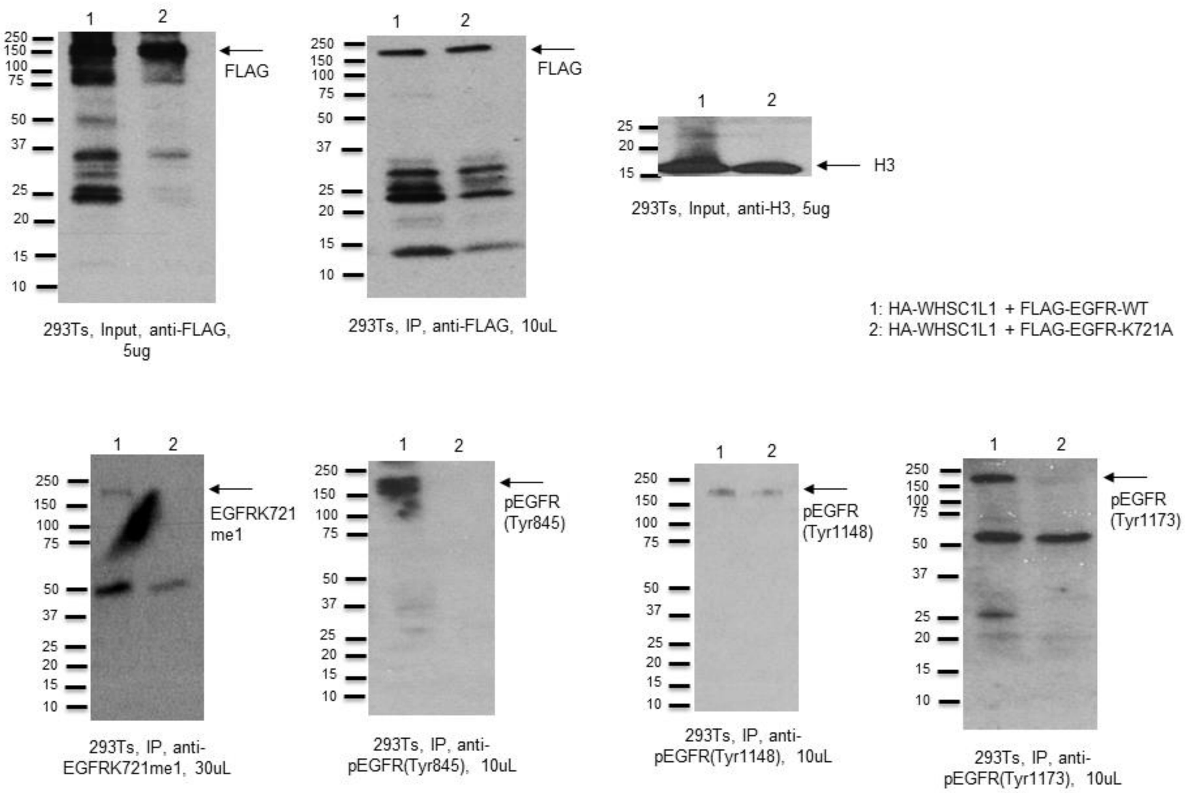

FLAG
293Ts, Input, anti-FLAG, 5ug
FLAG
293Ts, IP, anti-FLAG, 10uL
H3
293Ts, Input, anti-H3, 5ug
1: HA-WHSC1L1 + FLAG-EGFR-WT
2: HA-WHSC1L1 + FLAG-EGFR-K721A
EGFRK721 me1
293Ts, IP, anti-EGFRK721me1, 30uL
pEGFR (Tyr845)
293Ts, IP, anti-pEGFR(Tyr845), 10uL
pEGFR (Tyr1148)
293Ts, IP, anti-pEGFR(Tyr1148), 10uL
pEGFR (Tyr1173)
293Ts, IP, anti-pEGFR(Tyr1173), 10uL

Figure 3b

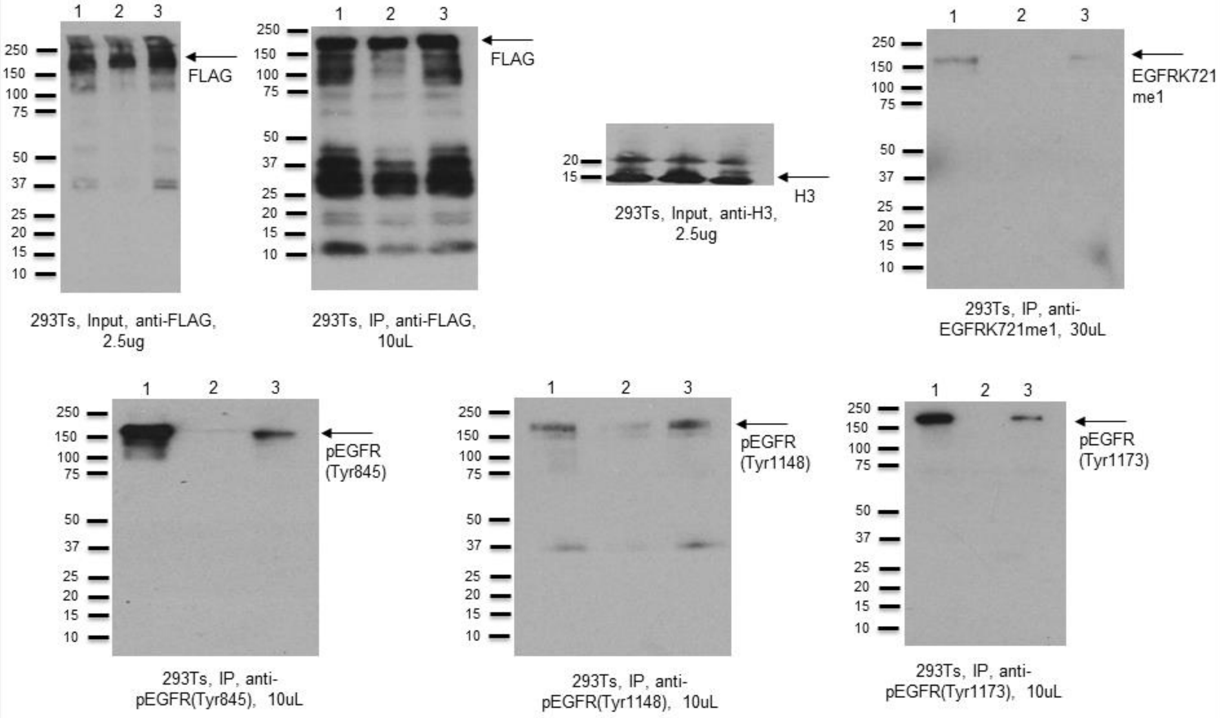

1: HA-WHSC1L1 + FLAG-EGFR-WT
2: HA-WHSC1L1 + FLAG-EGFR-K721A
3: HA-WHSC1L1 1-1073 + FLAG-EGFR-WT

Figure 3d

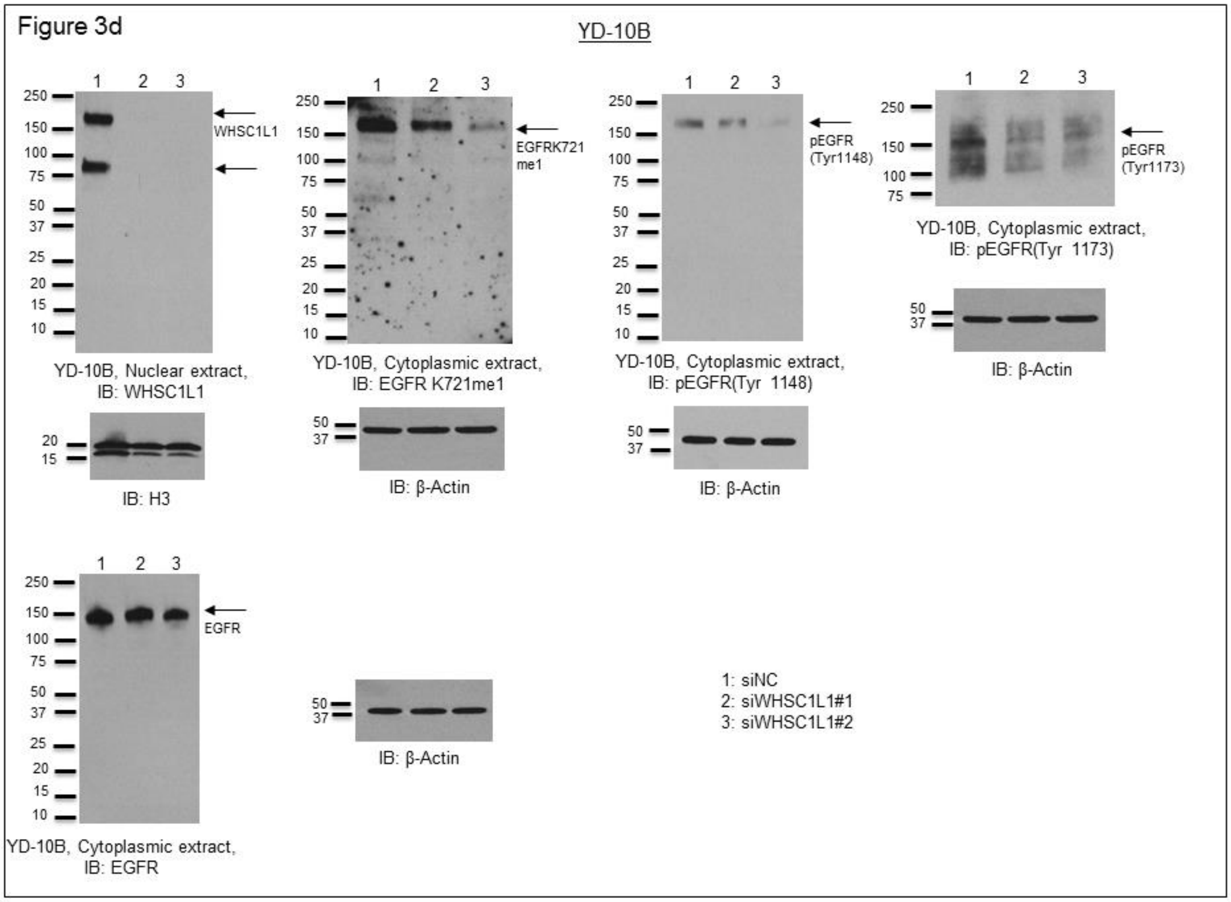
YD-10B
1 2 3
WHSC1L1
YD-10B, Nuclear extract,
IB: WHSC1L1
IB: H3
EGFRK721 me1
YD-10B, Cytoplasmic extract,
IB: EGFR K721me1
IB: β-Actin
pEGFR (Tyr1148)
YD-10B, Cytoplasmic extract,
IB: pEGFR(Tyr 1148)
IB: β-Actin
pEGFR (Tyr1173)
YD-10B, Cytoplasmic extract,
IB: pEGFR(Tyr 1173)
IB: β-Actin
EGFR
YD-10B, Cytoplasmic extract,
IB: EGFR
IB: β-Actin
1: siNC
2: siWHSC1L1#1
3: siWHSC1L1#2

Figure 3d (cont'ed)

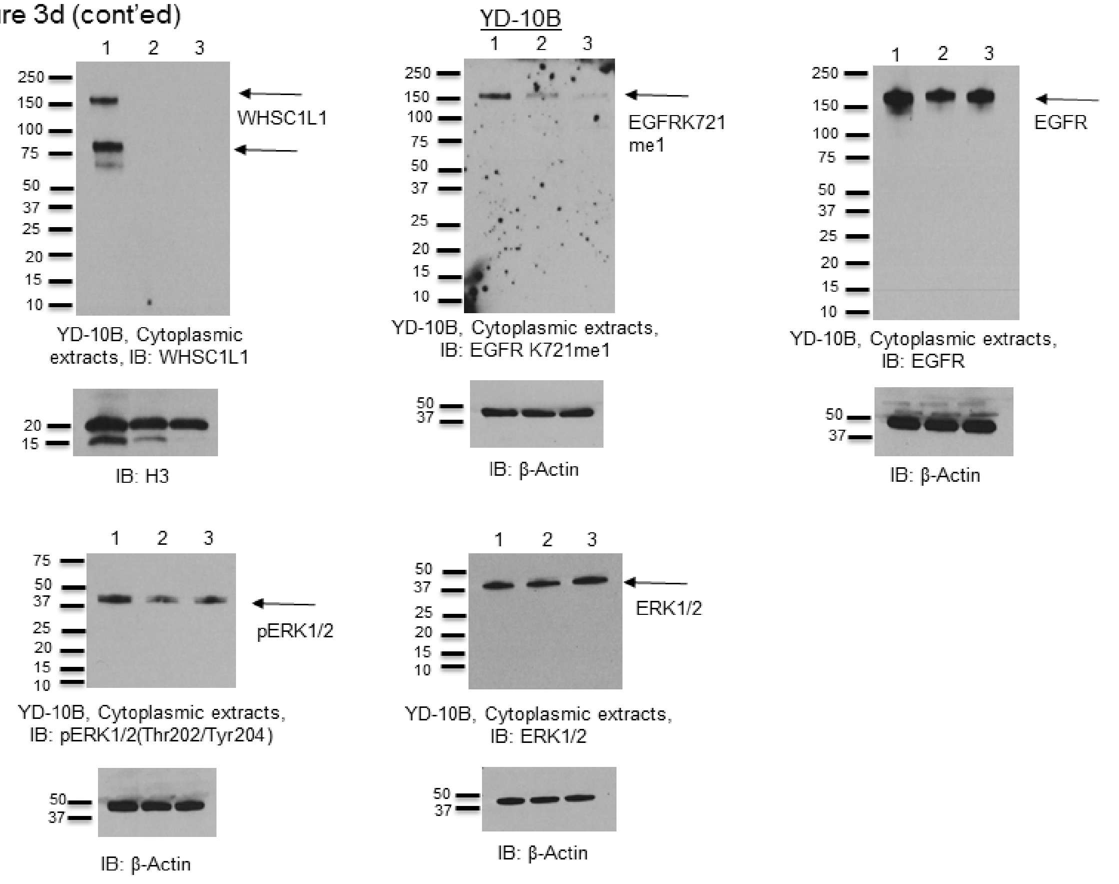

Figure 3e

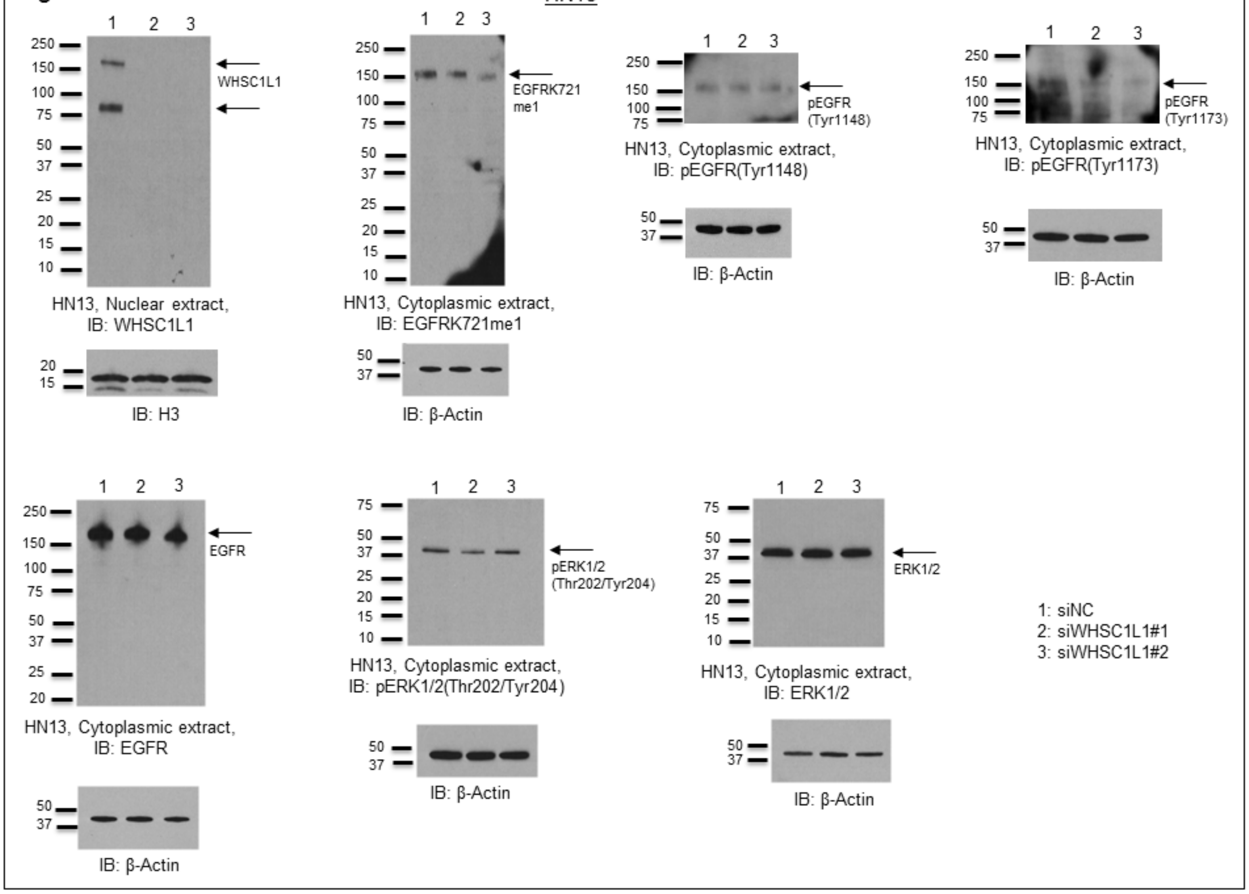
HN13
1 2 3
250
150
100
75
50
37
25
20
15
10
WHSC1L1
HN13, Nuclear extract,
IB: WHSC1L1
20
15
IB: H3
EGFRK721
me1
HN13, Cytoplasmic extract,
IB: EGFRK721me1
50
37
IB: β-Actin
pEGFR
(Tyr1148)
HN13, Cytoplasmic extract,
IB: pEGFR(Tyr1148)
IB: β-Actin
pEGFR
(Tyr1173)
HN13, Cytoplasmic extract,
IB: pEGFR(Tyr1173)
IB: β-Actin
EGFR
HN13, Cytoplasmic extract,
IB: EGFR
IB: β-Actin
pERK1/2
(Thr202/Tyr204)
HN13, Cytoplasmic extract,
IB: pERK1/2(Thr202/Tyr204)
IB: β-Actin
ERK1/2
HN13, Cytoplasmic extract,
IB: ERK1/2
IB: β-Actin
1: siNC
2: siWHSC1L1#1
3: siWHSC1L1#2

Figure 4a

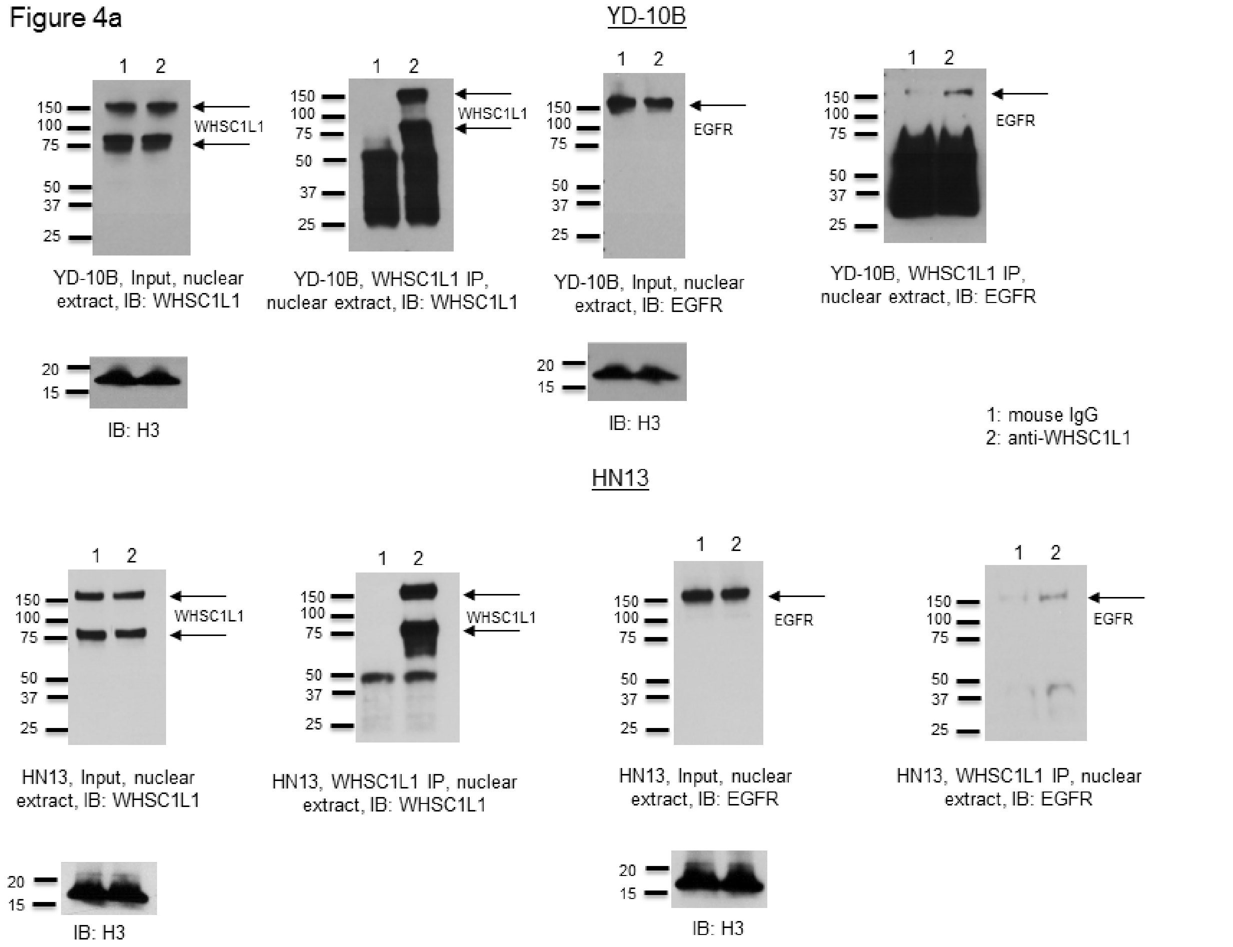
YD-10B
1 2
150
100
75
50
37
25
WHSC1L1
YD-10B, Input, nuclear extract, IB: WHSC1L1
YD-10B, WHSC1L1 IP, nuclear extract, IB: WHSC1L1
EGFR
YD-10B, Input, nuclear extract, IB: EGFR
YD-10B, WHSC1L1 IP, nuclear extract, IB: EGFR
20
15
IB: H3
1: mouse IgG
2: anti-WHSC1L1
HN13
HN13, Input, nuclear extract, IB: WHSC1L1
HN13, WHSC1L1 IP, nuclear extract, IB: WHSC1L1
HN13, Input, nuclear extract, IB: EGFR
HN13, WHSC1L1 IP, nuclear extract, IB: EGFR

Figure 4c

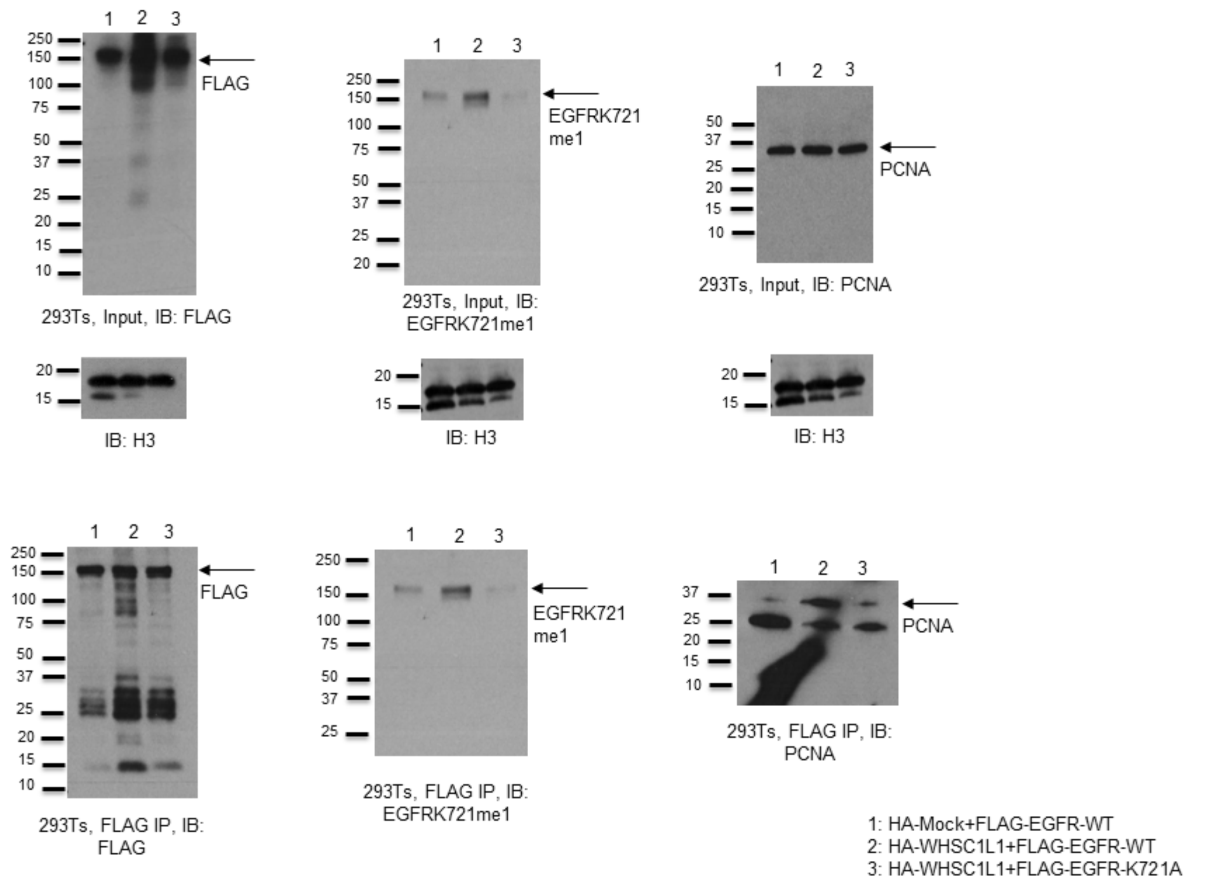
293Ts, Input, IB: FLAG
FLAG
293Ts, Input, IB: EGFRK721me1
EGFRK721 me1
293Ts, Input, IB: PCNA
PCNA
IB: H3
IB: H3
IB: H3
293Ts, FLAG IP, IB: FLAG
FLAG
293Ts, FLAG IP, IB: EGFRK721me1
EGFRK721 me1
293Ts, FLAG IP, IB: PCNA
PCNA
1: HA-Mock+FLAG-EGFR-WT
2: HA-WHSC1L1+FLAG-EGFR-WT
3: HA-WHSC1L1+FLAG-EGFR-K721A

Figure 4d

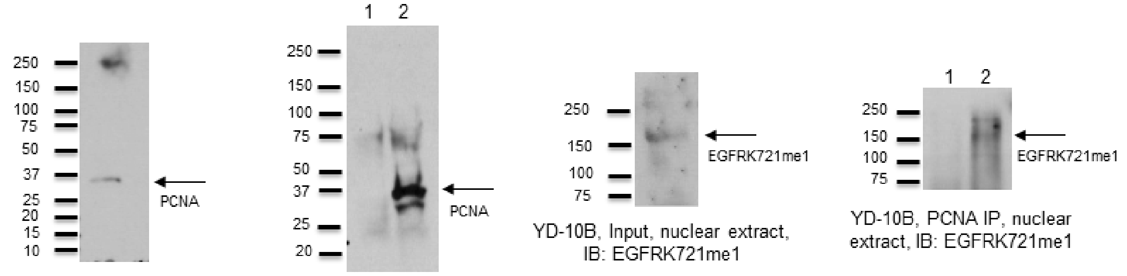

YD-10B, Input, nuclear extract, IB: PCNA

YD-10B, PCNA IP, nuclear extract, IB: PCNA

YD-10B, Input, nuclear extract, IB: EGFRK721me1

YD-10B, PCNA IP, nuclear extract, IB: EGFRK721me1

1: mouse IgG
2: anti-PCNA

Figure 4e

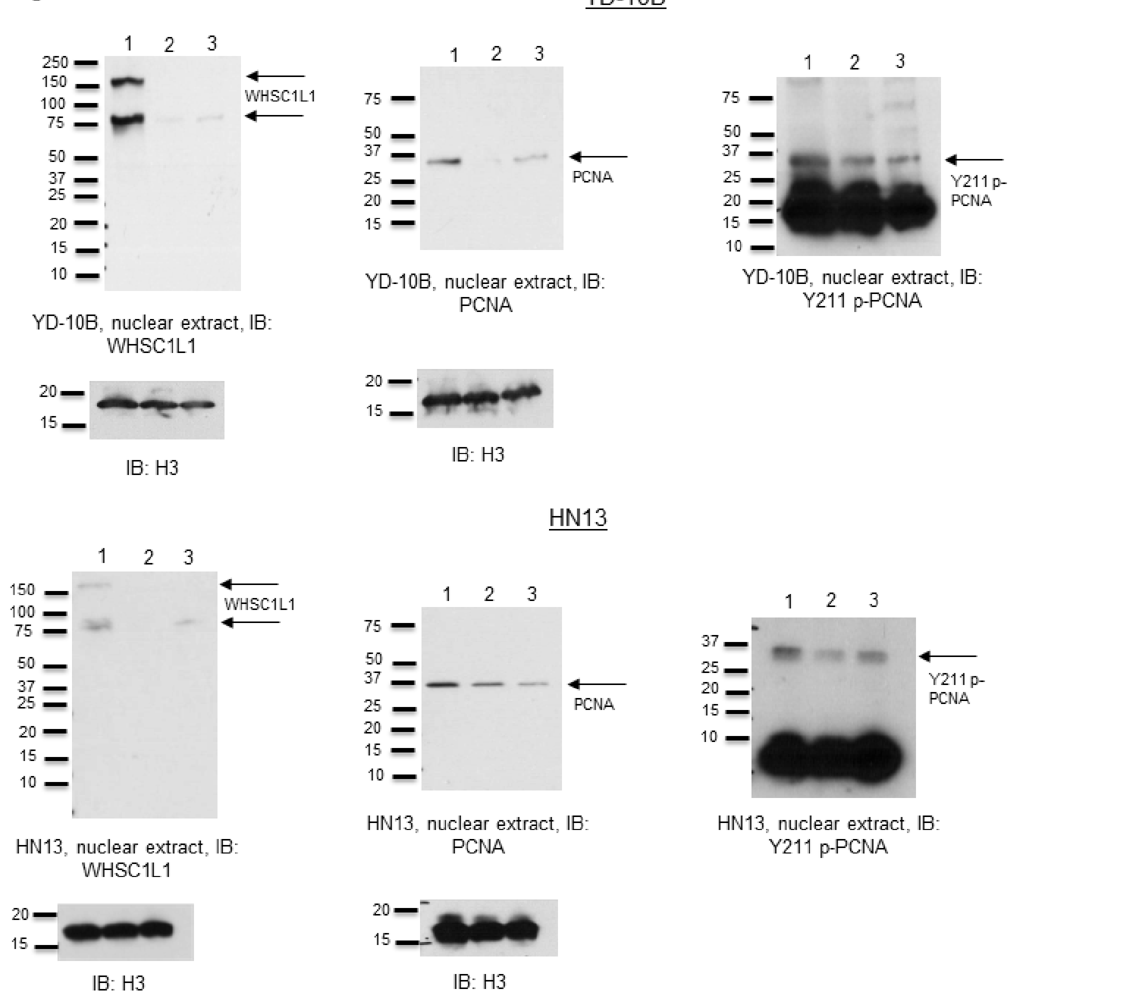
YD-10B
1 2 3
250
150
100
75
50
37
25
20
15
10
WHSC1L1
YD-10B, nuclear extract, IB: WHSC1L1
20
15
IB: H3
75
50
37
25
20
15
PCNA
YD-10B, nuclear extract, IB: PCNA
IB: H3
Y211 p-PCNA
YD-10B, nuclear extract, IB: Y211 p-PCNA
HN13
HN13, nuclear extract, IB: WHSC1L1
HN13, nuclear extract, IB: PCNA
HN13, nuclear extract, IB: Y211 p-PCNA
IB: H3
